# Global skin cancer burden and United States mortality trends in younger adults (Aged 20–44 Years): An analysis of the Global Burden of Disease Study 2021 and CDC WONDER Databases

**DOI:** 10.1016/j.xjidi.2026.100498

**Published:** 2026-06-04

**Authors:** Zhiqiang Ma, Pingyu An, Shidi Wu, Remco van Doorn, Maarten H. Vermeer, Abdoelwaheb El Ghalbzouri

**Affiliations:** 1 Department of Dermatology, Leiden University Medical Center, Leiden, The Netherlands; 2 Department of Pediatrics, The Second Affiliated Hospital of Harbin Medical University, Harbin, China; 3 Department of Dermatology, Netherlands Cancer Institute, Amsterdam, The Netherlands

**Keywords:** Basal cell carcinoma, Cutaneous T-cell lymphoma (CTCL), Epidemiology, Melanoma, Squamous cell carcinoma

## Abstract

Skin cancer is a growing public health concern, yet patterns in younger adults remain inadequately described. We quantified the global incidence, mortality, disability-adjusted life years, and age-standardized rates of melanoma and nonmelanoma skin cancer in adults aged 20–44 years using Global Burden of Disease 2021 estimates and assessed United States melanoma mortality in those aged 25–44 years from 1999 to 2023 using national vital statistics. From 1990 to 2021, age-standardized melanoma incidence declined, whereas nonmelanoma skin cancer cases rose from 142,018 to 292,052, with an increasing age-standardized incidence of basal cell carcinoma. The burden was greatest in countries with higher sociodemographic development and peaked at ages 40–44 years; women experienced a higher incidence, but men had higher mortality. In the United States, melanoma mortality decreased from 0.99 to 0.47 deaths per 100,000 between 1999 and 2023, representing an average annual reduction of 3.56%. Projections suggest continued declines in melanoma but sustained increases in nonmelanoma skin cancer. These findings indicate a growing and uneven skin cancer burden in young adults, supporting targeted prevention and earlier detection.

## Introduction

Skin cancer, primarily including basal cell carcinoma (BCC), squamous cell carcinoma (SCC), and melanoma, represents a substantial global public health burden (Fitzmaurice et al, 2019, Garbe et al, 2024). The etiology is multifactorial, with UVR, genetic susceptibility, and immunosuppression among the best-established risk factors (Cullen et al, 2020, Dummer et al, 2021, Long et al, 2023). In 2021, there were approximately 4.4 million new cases of BCC, 1.9 million of SCC, and 0.3 million of melanoma worldwide (Aggarwal et al, 2021, Zhou et al, 2025). Although the disease burden is well-characterized in older populations, its epidemiology in younger adults remains overlooked, and a systematic global assessment for this age group is lacking (Close et al, 2019, Miller et al, 2020). This study aimed to comprehensively evaluate the skin cancer burden—including prevalence, incidence, mortality, and disability-adjusted life years (DALYs)—among adults aged 20–44 years across 204 countries and territories, using data from the Global Burden of Disease (GBD) 2021 study. To address limitations of the GBD, particularly the absence of race or ethnicity data and information on other cutaneous malignancies (eg, cutaneous T-cell lymphoma), we also examined data from the Centers for Disease Control and Prevention Wide-ranging Online Data for Epidemiologic Research (CDC WONDER) database.

## Results

### Temporal trends and spatial distribution of skin cancer in younger adults

#### Melanoma

**Figure 1:**
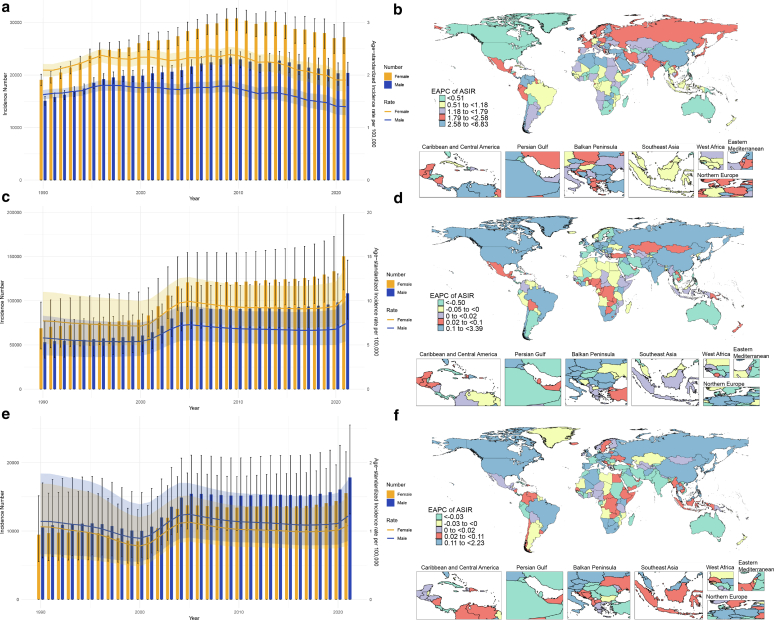
**Incidence trends and geographical distribution of skin cancers among individuals aged 20–44 years, 1990–2021.** (**a, b)** Malignant melanoma, (**c, d**) basal cell carcinoma, (**e, f**) squamous cell carcinoma. Panels **a**, **c**, and **e** show global incidence numbers by sex (bars) and age-standardized incidence rates (lines), with shaded areas indicating 95% uncertainty intervals. Panels **b**, **d**, and **f** show country- and territory-level EAPCs of ASIR from 1990 to 2021. ASIR, age-standardized incidence rate; EAPC, estimated annual percentage change.

**Figure 2:**
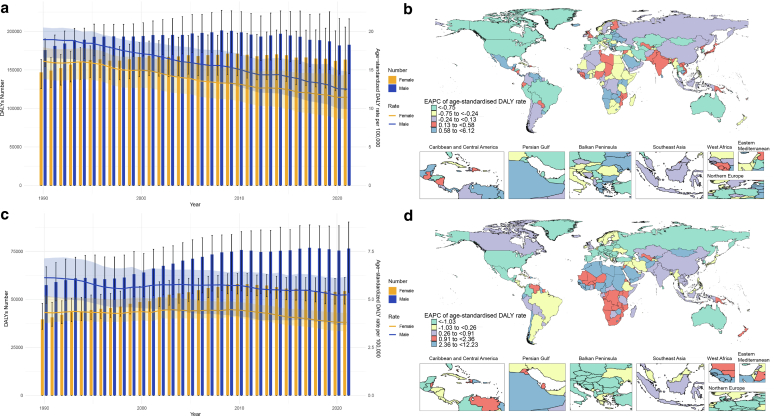
**DALY trends and geographical distribution of skin cancers among individuals aged 20–44 years, 1990–2021.** (**a, b**) Malignant melanoma and (**c, d**) squamous cell carcinoma. Panels **a** and **c** show global DALY numbers by sex (bars) and age-standardized DALY rates (lines), with shaded areas indicating 95% uncertainty intervals. Panels **b** and **d** show country- and territory-level EAPCs of the age-standardized DALY rate from 1990 to 2021. DALY, disability-adjusted life year; EAPC, estimated annual percentage change.

**Figure 3:**
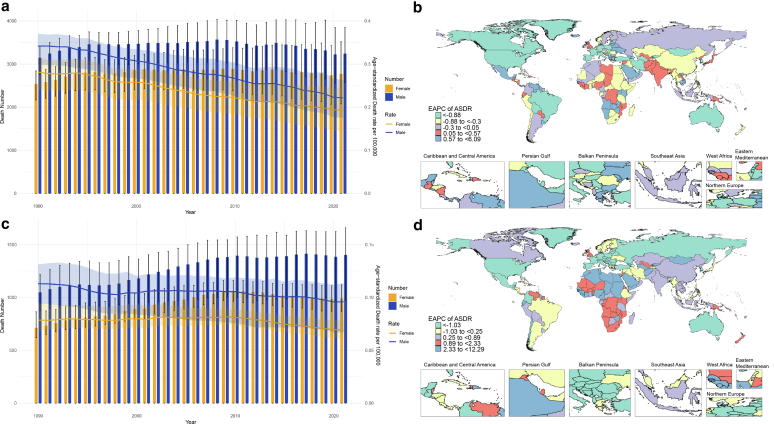
**Death trends and geographical distribution of skin cancers among individuals aged 20–44 years, 1990–2021.** (**a, b**) Malignant melanoma and (**c, d**) squamous cell carcinoma. Panels **a** and **c** show global death numbers by sex (bars) and age-standardized death rates (lines), with shaded areas indicating 95% uncertainty intervals. Panels **b** and **d** show country- and territory-level EAPCs of ASDR from 1990 to 2021. ASDR, age-standardized death rate; EAPC, estimated annual percentage change.

Globally, among adults aged 20–44 years, incident melanoma cases increased from 34,195 (95% uncertainty interval = 32,704–35,505) in 1990 to 47,634 (95% uncertainty interval = 42,754–50,867) in 2021. Although cases increased, the age-standardized incidence rate (ASIR) declined from 1.86 to 1.65 per 100,000 population (estimated annual percentage change [EAPC] = −0.37%; 95% confidence interval [CI] = −0.58% to −0.15%). Age-standardized rates (ASRs) for DALYs and mortality also declined (EAPC = −1.27% and −1.37%, respectively) (Table 1). Women had more incident cases, whereas men had higher DALY and mortality burdens. Across Sociodemographic Index (SDI) quintiles, ASIR, DALY, and mortality rates decreased in high-SDI regions but increased in all other quintiles. Nevertheless, high-SDI regions continued to have the highest burden in 2021. At the national level, the United States had the most incident cases. New Zealand had the highest 2021 ASIR, whereas Mauritius showed the fastest increase in ASIR from 1990 to 2021 (the highest EAPC), although from a low absolute level (Figure 1, Figure 2, Figure 3 and Table 1, Table 2, Table 3).

**Table 1:** Global Incidence, Prevalence, DALY, Death Numbers, ASRs in 1990 and 2021, and EAPCs of Skin Cancer

Disease	Case Number (95% UI)		ASR (per 100,000) (95% UI)		1990–2021 EAPC (95% CI)
	1990	2021	1990	2021	
Malignant skin melanoma					
Incidence	34,195 (32,704–35,505)	47,634 (42,754–50,867)	1.86 (1.78–1.93)	1.65 (1.48–1.76)	−0.37 (−0.58 to −0.15)
Prevalence	277,782 (267,521–287,707)	399,678 (364,763–423,968)	15.06 (14.51–15.59)	13.83 (12.62–14.67)	−0.24 (−0.48 to 0.01)
DALYs	322,467 (288,008–344,308)	345,956 (277,854–400,375)	17.55 (15.7–18.73)	11.97 (9.61–13.86)	−1.27 (−1.31 to −1.23)
Deaths	5681 (5045–6063)	6013 (4817–6952)	0.31 (0.28–0.33)	0.21 (0.17–0.24)	−1.37 (−1.41 to −1.33)
NMSC					
Incidence	142,018 (99,160–197,469)	292,052 (223,009–377,723)	7.84 (5.5–10.86)	10.07 (7.68–13.03)	0.89 (0.61–1.18)
Prevalence	41,634 (28,250–60,582)	75,213 (56,031–104,326)	2.26 (1.55–3.28)	2.6 (1.93–3.6)	0.42 (0.16–0.68)
DALYs	97,099 (85,994–110,962)	130,945 (105,210–148,279)	5.24 (4.64–5.98)	4.53 (3.64–5.13)	−0.36 (−0.45 to −0.27)
Deaths	1761 (1559–2010)	2394 (1923–2713)	0.1 (0.09–0.11)	0.08 (0.07–0.09)	−0.37 (−0.46 to −0.28)
NMSC (BCC)					
Incidence	122,043 (80,618–174,850)	258,700 (191,842–342,976)	6.74 (4.48–9.62)	8.92 (6.61–11.83)	0.99 (0.71–1.27)
Prevalence	16,417 (10,227–25,558)	33,541 (22,520–49,944)	0.9 (0.57–1.4)	1.16 (0.78–1.72)	0.75 (0.53–0.98)
DALYs	72 (28–148)	146 (59–291)	0.004 (0.002–0.008)	0.005 (0.002–0.01)	0.7 (0.48–0.92)
NMSC (SCC)					
Incidence	19,975 (11,645–32,235)	33,352 (23,782–47,084)	1.1 (0.65–1.77)	1.15 (0.82–1.62)	0.25 (−0.07 to 0.57)
Prevalence	25,217 (14,054–41,321)	41,673 (27,335–64,772)	1.36 (0.76–2.22)	1.44 (0.94–2.24)	0.17 (−0.12 to 0.47)
DALYs	97,026 (85,943–110,894)	130,799 (105,075–148,097)	5.24 (4.64–5.98)	4.53 (3.64–5.13)	−0.36 (−0.45 to −0.27)
Deaths	1761 (1559–2010)	2394 (1923–2713)	0.1 (0.09–0.11)	0.08 (0.07–0.09)	−0.37 (−0.46 to −0.28)

For BCC and SCC, GBD 2021 relies partly on statistical modeling because many cancer registries do not routinely collect or code these histological subtypes. As a result, subtype-specific NMSC estimates—particularly in low- and middle-SDI regions—have wide UIs and are more reliable at the global or regional level than for individual countries. GBD age-standardized rates were calculated using the GBD standard population structure.

Abbreviations: ASR, age-standardized rate; BCC, basal cell carcinoma; CI, confidence interval; DALY, disability-adjusted life year; EAPC, estimated annual percentage change; GBD, Global Burden of Disease; NMSC, nonmelanoma skin cancer; SCC, squamous cell carcinoma; SDI, Sociodemographic Index; UI, uncertainty interval.

**Table 2:** Incidence, Prevalence, DALY, Death Numbers, ASRs in 2021, and EAPCs of Malignant Melanoma for Different SDI and GBD Regions

Location	Incidence			Prevalence			DALYs			Deaths		
	Case Number (95% UI)	ASR (per 100,000) (95% UI)	1990–2021 EAPC (95% CI)	Case Number (95% UI)	ASR (per 100,000) (95% UI)	1990–2021 EAPC (95% CI)	Case Number (95% UI)	ASR (per 100,000) (95% UI)	1990–2021 EAPC (95% CI)	Case Number (95% UI)	ASR (per 100,000) (95% UI)	1990–2021 EAPC (95% CI)
SDI												
High SDI	27,825 (26,529–29,224)	7.11 (6.78–7.47)	−0.06 (−0.39 to 0.27)	249,202 (237,440–261,825)	63.76 (60.74–66.99)	0.04 (−0.3 to 0.39)	101,762 (95,987–108,537)	25.76 (24.29–27.49)	−1.69 (−1.83 to −1.55)	1650 (1591–1707)	0.41 (0.4–0.43)	−1.92 (−2.05 to −1.79)
High-middle SDI	11,255 (9808–12,365)	2.26 (1.97–2.48)	1.64 (1.4–1.87)	95,489 (83,304–105,137)	19.21 (16.74–21.16)	2.12 (1.83–2.4)	82,387 (71,202–91,120)	16.37 (14.15–18.13)	−0.71 (−0.83 to −0.59)	1455 (1261–1609)	0.29 (0.25–0.32)	−0.85 (−0.96 to −0.73)
Middle SDI	5040 (3631–6064)	0.54 (0.39–0.65)	2.1 (2.03–2.17)	37,014 (26,650–44,624)	3.95 (2.85–4.77)	3.82 (3.73–3.9)	69,757 (51,004–83,658)	7.42 (5.43–8.9)	−0.33 (−0.37 to −0.28)	1260 (921–1509)	0.13 (0.1–0.16)	−0.41 (−0.46 to −0.36)
Low-middle SDI	2074 (1304–2692)	0.29 (0.18–0.38)	1.85 (1.78–1.92)	11,221 (7151–14,588)	1.56 (1–2.03)	3.24 (3.13–3.35)	49,694 (31,591–66,629)	6.98 (4.45–9.35)	0.71 (0.67–0.75)	894 (572–1196)	0.13 (0.08–0.17)	0.68 (0.64–0.72)
Low SDI	1389 (796–1948)	0.4 (0.23–0.56)	0.78 (0.66–0.89)	6320 (3547–9144)	1.75 (0.99–2.53)	2.02 (1.82–2.23)	41,951 (24,211–58,416)	11.98 (6.93–16.65)	0.01 (−0.07 to 0.09)	747 (432–1038)	0.22 (0.13–0.3)	−0.01 (−0.09 to 0.07)
GBD regions												
High-income Asia Pacific	928 (732–1135)	1.59 (1.25–1.95)	2.88 (2.39–3.36)	8379 (6606–10,250)	14.33 (11.29–17.6)	3.04 (2.54–3.55)	3256 (2708–3783)	5.46 (4.55–6.36)	−0.09 (−0.3 to 0.12)	52 (44–59)	0.09 (0.07–0.1)	−0.43 (−0.62 to −0.23)
High-income North America	11,983 (11,340–12,660)	9.43 (8.92–9.96)	−1.19 (−1.5 to −0.88)	107,532 (101,788–113,630)	84.64 (80.11–89.45)	−1.12 (−1.45 to −0.8)	38,345 (35,919–41,295)	30.03 (28.13–32.36)	−2.45 (−2.58 to −2.31)	606 (582–632)	0.47 (0.45–0.49)	−2.68 (−2.79 to −2.56)
Western Europe	14,865 (13,738–15,996)	10.36 (9.57–11.15)	1.82 (1.35–2.3)	133,916 (123,773–144,190)	93.37 (86.25–100.56)	1.96 (1.47–2.45)	53,975 (50,437–58,169)	37.15 (34.7–40.06)	−0.54 (−0.77 to −0.31)	876 (834–919)	0.59 (0.57–0.62)	−0.82 (−1.03 to −0.6)
Australasia	2241 (1790–2781)	20.2 (16.12–25.08)	−0.36 (−0.85 to 0.12)	20,247 (16,164–25,114)	182.47 (145.63–226.54)	−0.28 (−0.78 to 0.21)	7648 (6467–8934)	68.77 (58.12–80.38)	−2.39 (−2.65 to −2.13)	122 (105–140)	1.09 (0.93–1.25)	−2.63 (−2.86 to −2.39)
Andean Latin America	149 (103–226)	0.59 (0.41–0.9)	1.91 (1.76–2.05)	1062 (724–1646)	4.21 (2.87–6.52)	4.21 (4.01–4.41)	2425 (1723–3531)	9.65 (6.86–14.05)	−0.44 (−0.56 to −0.32)	44 (31–64)	0.18 (0.12–0.26)	−0.5 (−0.61 to −0.38)
Tropical Latin America	1069 (980–1171)	1.16 (1.06–1.27)	0.86 (0.63–1.08)	7754 (7080–8536)	8.46 (7.72–9.31)	2.09 (1.85–2.33)	17,151 (15,972–18,350)	18.56 (17.28–19.85)	−0.82 (−1.02 to −0.62)	312 (291–334)	0.34 (0.31–0.36)	−0.88 (−1.09 to −0.68)
Central Latin America	728 (635–828)	0.76 (0.66–0.86)	2.47 (2.31–2.64)	5468 (4738–6281)	5.7 (4.94–6.54)	3.82 (3.6–4.04)	10,782 (9592–11,975)	11.22 (9.98–12.46)	0.52 (0.4–0.64)	194 (172–215)	0.2 (0.18–0.22)	0.45 (0.33–0.57)
Southern Latin America	536 (446–645)	2.08 (1.73–2.5)	2.19 (1.79–2.58)	4410 (3658–5322)	17.1 (14.19–20.65)	2.97 (2.5–3.44)	5532 (4765–6407)	21.39 (18.42–24.78)	0.05 (−0.27 to 0.37)	98 (85–113)	0.38 (0.33–0.44)	−0.06 (−0.38 to 0.26)
Caribbean	96 (77–120)	0.56 (0.45–0.69)	0.58 (0.3–0.86)	682 (554–826)	3.95 (3.21–4.79)	0.92 (0.63–1.22)	1631 (1199–2282)	9.47 (6.97–13.25)	−0.05 (−0.29 to 0.19)	29 (22–41)	0.17 (0.13–0.24)	−0.06 (−0.31 to 0.18)
Central Europe	2152 (1872–2482)	5.12 (4.45–5.91)	2.41 (2.2–2.63)	18,197 (15,786–21,063)	43.46 (37.67–50.37)	3.18 (2.89–3.47)	16,889 (15,094–18,888)	39.49 (35.27–44.18)	−0.54 (−0.66 to −0.43)	300 (268–333)	0.69 (0.61–0.76)	−0.73 (−0.85 to −0.6)
Eastern Europe	3651 (3292–4007)	4.52 (4.07–4.96)	2.44 (2.13–2.75)	29,504 (26,618–32,515)	36.73 (33.1–40.5)	2.92 (2.59–3.26)	34,871 (31,053–38,954)	42.7 (38.03–47.74)	0.34 (0.03 to 0.65)	629 (560–703)	0.76 (0.67–0.85)	0.28 (−0.04 to 0.59)
Central Asia	177 (147–213)	0.48 (0.4–0.58)	−0.45 (−0.79 to −0.12)	1290 (1071–1562)	3.48 (2.89–4.21)	0.27 (−0.16 to 0.7)	2972 (2510–3545)	8.06 (6.81–9.61)	−1.73 (−1.94 to −1.52)	53 (45–64)	0.14 (0.12–0.17)	−1.75 (−1.96 to −1.55)
North Africa and Middle East	2383 (1282–3074)	0.95 (0.51–1.23)	2.12 (1.98–2.26)	19,300 (10,346–25,175)	7.72 (4.14–10.07)	3.24 (3.04–3.45)	9518 (5083–12,078)	3.8 (2.03–4.82)	−1.13 (−1.16 to −1.1)	155 (82–197)	0.06 (0.03–0.08)	−1.41 (−1.44 to −1.38)
South Asia	1676 (1025–2494)	0.23 (0.14–0.35)	1.84 (1.71–1.96)	9371 (5582–14,138)	1.3 (0.78–1.96)	3.51 (3.34–3.68)	39,404 (24,905–57,658)	5.52 (3.5–8.06)	0.41 (0.34–0.47)	706 (450–1030)	0.1 (0.06–0.14)	0.37 (0.31–0.43)
Southeast Asia	348 (241–491)	0.13 (0.09–0.18)	0.81 (0.73–0.89)	1447 (982–2060)	0.53 (0.36–0.76)	2.22 (2.09–2.36)	10,279 (7190–14,345)	3.75 (2.62–5.23)	−0.05 (−0.14 to 0.03)	189 (132–264)	0.07 (0.05–0.1)	−0.05 (−0.14 to 0.03)
East Asia	2785 (1520–3836)	0.51 (0.28–0.71)	3.36 (3.04–3.67)	22,329 (12,042–31,046)	4.12 (2.22–5.74)	6.09 (5.87–6.31)	36,332 (20,423–48,628)	6.68 (3.75–8.94)	−0.2 (−0.35 to −0.05)	662 (371–882)	0.12 (0.07–0.16)	−0.31 (−0.46 to −0.16)
Oceania	3 (2–5)	0.06 (0.04–0.1)	0.01 (−0.07 to 0.09)	8 (5–12)	0.16 (0.1–0.25)	0.18 (0.01–0.36)	125 (79–191)	2.54 (1.61–3.86)	−0.06 (−0.12 to 0.01)	2 (1–3)	0.05 (0.03–0.07)	−0.07 (−0.13 to 0)
Western Sub-Saharan Africa	476 (145–743)	0.33 (0.1–0.51)	1.01 (0.88–1.15)	2103 (610–3369)	1.42 (0.41–2.26)	2.19 (1.96–2.42)	13,425 (4364–20,181)	9.35 (3.05–14)	0.13 (0.04–0.23)	241 (79–361)	0.17 (0.06–0.25)	0.11 (0.01–0.2)
Eastern Sub-Saharan Africa	975 (537–1549)	0.73 (0.4–1.15)	0.75 (0.62–0.87)	4610 (2445–7618)	3.32 (1.77–5.45)	1.95 (1.7–2.2)	30,090 (16,733–46,281)	22.42 (12.52–34.31)	0.08 (0–0.15)	534 (298–817)	0.41 (0.23–0.62)	0.06 (−0.02 to 0.13)
Central Sub-Saharan Africa	103 (63–185)	0.25 (0.15–0.45)	0.86 (0.62–1.09)	464 (277–865)	1.1 (0.66–2.03)	2.19 (1.77–2.61)	3333 (2043–5804)	8.11 (4.98–14.06)	0.25 (0.1–0.4)	61 (37–105)	0.15 (0.09–0.26)	0.24 (0.09 to 0.39)
Southern Sub-Saharan Africa	311 (191–470)	0.99 (0.61–1.49)	1.06 (0.72–1.40)	1606 (967–2606)	5.04 (3.03–8.15)	1.53 (1.16–1.91)	7974 (4862–11,468)	25.27 (15.4–36.32)	0.62 (0.29–0.95)	146 (90–210)	0.47 (0.28–0.67)	0.62 (0.31–0.94)

Abbreviations: CI, confidence interval; DALY, disability-adjusted life year; EAPC, estimated annual percentage change; GBD, Global Burden of Disease; SDI, Sociodemographic Index; UI, uncertainty interval.

**Table 3:** Incidence, Death, and DALY Numbers, and Age-Standardized Rates of Malignant Melanoma, across 204 Countries and Territories in 2021, Including EAPCs from 1990 to 2021

Location	Incidence			Death			Disability-Adjusted Life Year		
	Number	ASIR	EAPC	Number	ASDR	EAPC	Number	Age-Standardized DALY Rate	EAPC
Afghanistan	36.6	0.43	1.46	10.0	0.12	0.06	576.0	6.82	0.10
Albania	17.3	1.89	3.25	2.8	0.31	−0.25	158.6	17.44	−0.17
Algeria	75.2	0.42	3.06	5.8	0.03	−0.21	347.7	1.97	0.02
American Samoa	0.0213	0.13	0.79	0.0126	0.08	0.62	0.7	4.21	0.59
Andorra	3.4	10.54	1.07	0.2	0.53	−1.16	11.1	33.96	−0.88
Angola	26.3	0.29	1.67	15.0	0.17	0.82	830.0	9.01	0.86
Antigua and Barbuda	0.3	0.86	1.44	0.0713	0.20	0.05	4.0	11.25	0.10
Argentina	307.9	1.76	1.74	67.4	0.38	−0.06	3749.8	21.38	0.03
Armenia	3.2	0.27	1.90	0.7	0.06	−0.10	40.8	3.45	−0.08
Australia	1833.7	19.77	−0.61	97.9	1.05	−2.95	6168.2	66.31	−2.70
Austria	330.3	10.72	1.78	22.5	0.72	−0.70	1376.0	44.22	−0.46
Azerbaijan	13.4	0.31	0.25	4.3	0.10	−1.36	239.8	5.47	−1.33
Bahamas	2.0	1.32	1.53	0.7	0.44	0.46	36.5	24.08	0.48
Bahrain	2.7	0.35	3.16	0.2	0.02	−0.67	9.8	1.27	−0.32
Bangladesh	140.1	0.22	1.75	51.6	0.08	−0.33	2916.6	4.65	−0.26
Barbados	0.7	0.64	1.21	0.2	0.15	−0.12	9.0	8.69	−0.10
Belarus	165.8	4.61	4.26	24.9	0.68	1.68	1391.6	38.34	1.78
Belgium	419.5	10.96	1.95	26.4	0.67	−0.36	1604.2	41.48	−0.15
Belize	0.6	0.40	2.80	0.2	0.14	1.68	12.4	7.72	1.70
Benin	4.7	0.12	0.32	2.4	0.06	−0.45	135.7	3.50	−0.44
Bermuda	1.0	4.82	2.21	0.1	0.49	−1.02	6.2	28.68	−0.84
Bhutan	0.7	0.20	1.32	0.3	0.08	−0.43	15.0	4.64	−0.38
Bolivia (Plurinational State of)	27.2	0.61	0.46	12.6	0.29	−0.93	688.3	15.56	−0.91
Bosnia and Herzegovina	35.9	3.12	2.94	5.9	0.49	−0.17	331.0	28.45	−0.04
Botswana	6.8	0.66	0.26	3.4	0.33	−0.60	182.6	17.89	−0.58
Brazil	1046.0	1.17	0.85	305.3	0.34	−0.90	16756.9	18.69	−0.84
Brunei Darussalam	1.5	0.72	1.92	0.4	0.19	0.34	22.4	10.53	0.33
Bulgaria	112.2	4.77	3.17	17.4	0.72	1.28	972.3	40.93	1.37
Burkina Faso	9.1	0.14	0.19	5.1	0.08	−0.37	281.2	4.30	−0.36
Burundi	24.7	0.60	−0.93	15.6	0.38	−1.39	880.3	21.24	−1.37
Cabo Verde	0.4	0.18	3.90	0.1	0.05	1.77	6.3	2.65	1.81
Cambodia	8.5	0.13	1.62	5.6	0.09	0.90	310.2	4.79	0.90
Cameroon	17.9	0.18	0.55	8.9	0.09	−0.18	493.4	4.95	−0.17
Canada	848.1	6.63	0.30	70.3	0.54	−1.61	4205.6	32.59	−1.41
Central African Republic	3.4	0.20	−0.20	2.5	0.15	−0.34	136.8	8.14	−0.33
Chad	4.8	0.11	0.82	3.0	0.07	0.44	165.3	3.82	0.45
Chile	186.0	2.61	3.06	23.4	0.33	−0.40	1353.9	19.01	−0.22
China	2716.3	0.52	3.44	629.6	0.12	−0.34	34603.7	6.60	−0.22
Colombia	206.8	1.08	2.63	42.0	0.22	−0.25	2378.1	12.49	−0.17
Comoros	2.0	0.74	−0.13	1.1	0.40	−0.84	60.5	22.38	−0.85
Congo	6.7	0.34	0.95	3.6	0.18	−0.04	194.1	9.98	0.00265
Cook Islands	0.00476	0.08	1.93	0.00173	0.03	0.33	0.0955	1.69	0.40
Costa Rica	33.7	1.77	1.61	6.1	0.32	−0.27	342.9	18.00	−0.20
Côte d’Ivoire	32.3	0.35	1.42	16.3	0.18	0.57	906.5	9.69	0.61
Croatia	71.2	4.89	2.08	10.2	0.69	−0.59	575.4	39.34	−0.47
Cuba	34.6	0.95	1.06	6.1	0.17	−0.50	342.8	9.37	−0.48
Cyprus	34.8	5.91	4.56	2.2	0.36	0.26	133.1	22.43	0.62
Czechia	437.3	12.06	3.15	27.4	0.72	−0.29	1671.3	44.99	0.12
Democratic People's Republic of Korea	24.6	0.24	0.51	16.7	0.16	0.20	896.9	8.62	0.19
Democratic Republic of the Congo	62.7	0.23	0.58	37.6	0.14	0.11	2071.4	7.63	0.11
Denmark	259.0	13.87	2.34	12.3	0.65	−1.14	786.8	42.01	−0.70
Djibouti	3.1	0.61	0.39	1.7	0.33	−0.29	92.0	18.12	−0.27
Dominica	0.0806	0.33	1.76	0.0327	0.14	1.14	1.8	7.48	1.17
Dominican Republic	8.7	0.21	1.22	3.4	0.08	0.15	186.0	4.42	0.18
Ecuador	42.9	0.64	2.30	13.2	0.20	0.39	725.2	10.90	0.45
Egypt	28.7	0.08	1.22	3.0	0.00799	−0.74	177.9	0.47	−0.62
El Salvador	6.2	0.27	3.35	1.6	0.07	0.81	92.0	4.05	0.89
Equatorial Guinea	1.9	0.37	2.17	0.8	0.16	0.00881	43.3	8.63	0.06
Eritrea	17.3	0.76	0.38	11.0	0.49	−0.06	609.4	26.59	−0.04
Estonia	29.2	6.32	2.14	2.9	0.60	−1.19	165.5	35.41	−0.96
Eswatini	3.5	0.86	1.18	1.9	0.48	0.84	105.3	25.65	0.83
Ethiopia	117.0	0.34	0.53	63.8	0.19	−0.58	3584.7	10.27	−0.54
Fiji	0.4	0.11	0.81	0.2	0.07	0.56	13.7	3.98	0.57
Finland	262.7	14.66	3.38	13.8	0.75	−0.16	874.2	48.24	0.29
France	2074.2	9.87	2.24	113.8	0.53	−1.07	7047.6	33.26	−0.75
Gabon	2.3	0.38	0.62	1.0	0.17	−0.53	57.2	9.39	−0.52
Gambia	1.5	0.20	1.33	0.8	0.10	0.61	42.4	5.61	0.64
Georgia	13.6	1.07	−2.17	4.0	0.31	−2.34	217.9	17.17	−2.43
Germany	2699.7	9.58	1.17	148.4	0.52	−1.26	9230.9	32.45	−1.01
Ghana	2.6	0.02	0.42	1.2	0.01	−0.37	67.2	0.56	−0.36
Greece	218.9	6.43	2.00	16.7	0.47	0.88	981.9	28.14	0.96
Greenland	0.3	1.35	−0.14	0.0729	0.37	−1.76	4.0	19.92	−1.75
Grenada	0.2	0.67	0.80	0.0831	0.23	−0.28	4.6	12.74	−0.27
Guam	0.0192	0.04	0.84	0.00904	0.02	0.63	0.5	0.94	0.57
Guatemala	16.9	0.30	1.83	6.9	0.13	0.28	380.9	6.85	0.30
Guinea	22.5	0.58	0.89	13.9	0.36	0.47	777.0	19.86	0.50
Guinea-Bissau	1.2	0.18	0.45	0.7	0.11	−0.09	39.1	6.04	−0.08
Guyana	0.9	0.35	2.39	0.5	0.18	1.80	26.2	9.68	1.78
Haiti	17.5	0.36	0.50	11.7	0.24	0.05	638.7	13.12	0.06
Honduras	6.2	0.17	0.26	2.7	0.08	−0.79	148.1	4.17	−0.81
Hungary	186.8	5.62	0.94	23.4	0.67	−1.85	1329.0	39.24	−1.65
Iceland	22.7	18.12	4.18	1.2	0.95	2.05	76.2	60.61	2.32
India	1155.5	0.21	1.81	465.8	0.08	0.22	25775.3	4.64	0.24
Indonesia	102.9	0.09	0.59	65.0	0.06	0.01	3530.0	3.10	0.01
Iran (Islamic Republic of)	890.3	2.32	1.79	35.5	0.09	−1.74	2344.8	6.02	−1.29
Iraq	78.4	0.52	2.66	6.6	0.04	−0.71	393.8	2.62	−0.50
Ireland	230.9	13.07	3.41	11.9	0.64	−0.26	742.0	41.05	0.13
Israel	308.7	9.60	1.95	23.1	0.71	−1.02	1397.7	43.21	−0.75
Italy	1931.6	10.61	1.33	107.5	0.56	−1.00	6640.3	35.53	−0.75
Jamaica	5.7	0.52	2.20	1.6	0.15	1.34	89.8	8.24	1.33
Japan	619.9	1.68	2.44	33.8	0.09	0.05	2121.1	5.63	0.33
Jordan	17.2	0.36	1.87	1.0	0.02	−1.82	61.9	1.31	−1.46
Kazakhstan	73.3	1.01	1.57	17.7	0.24	−0.80	980.8	13.48	−0.79
Kenya	73.7	0.43	1.32	33.7	0.20	0.86	1895.5	11.12	0.87
Kiribati	0.00882	0.02	0.59	0.0069	0.02	0.44	0.4	0.88	0.45
Kuwait	6.4	0.24	−1.10	0.3	0.00939	−2.48	17.0	0.63	−2.23
Kyrgyzstan	12.1	0.49	−2.48	3.8	0.16	−3.91	212.9	8.60	−3.89
Lao People's Democratic Republic	3.4	0.12	0.64	2.4	0.09	0.09	133.0	4.71	0.13
Latvia	29.8	4.66	2.29	5.4	0.83	0.75	296.6	45.98	0.79
Lebanon	26.4	1.11	2.67	1.3	0.05	−1.00	83.1	3.49	−0.59
Lesotho	4.1	0.62	1.92	2.6	0.40	1.90	140.8	21.49	1.91
Liberia	2.9	0.16	1.42	1.4	0.08	0.22	78.2	4.18	0.24
Libya	10.8	0.34	2.73	1.0	0.03	0.22	56.7	1.78	0.42
Lithuania	42.0	4.68	2.71	7.1	0.79	1.53	392.9	43.70	1.56
Luxembourg	22.4	8.86	1.01	1.2	0.48	−2.26	77.2	30.08	−1.96
Madagascar	59.1	0.65	−0.07	34.1	0.38	−0.55	1925.5	21.02	−0.53
Malawi	104.2	1.71	1.09	56.0	0.94	0.36	3187.7	52.21	0.38
Malaysia	30.5	0.23	1.04	14.1	0.11	−0.20	773.6	5.89	−0.22
Maldives	0.2	0.06	2.38	0.0705	0.02	0.20	3.8	1.32	0.24
Mali	36.3	0.58	0.53	19.7	0.32	−0.13	1112.1	17.58	−0.10
Malta	11.5	7.19	4.15	0.8	0.47	1.50	46.0	28.35	1.69
Marshall Islands	0.016	0.07	0.54	0.0118	0.05	0.33	0.6	2.93	0.34
Mauritania	2.0	0.16	1.28	0.8	0.06	−0.32	44.4	3.46	−0.30
Mauritius	0.8	0.16	6.83	0.4	0.08	6.09	20.3	4.10	6.12
Mexico	369.7	0.75	2.50	108.9	0.22	0.74	6028.5	12.20	0.80
Micronesia (Federated States of)	0.0256	0.07	0.17	0.0179	0.05	−0.25	1.0	2.74	−0.24
Monaco	1.6	15.87	2.53	0.0971	0.90	0.90	6.0	56.92	1.08
Mongolia	3.3	0.26	0.17	1.3	0.10	−1.70	69.5	5.35	−1.70
Montenegro	10.1	4.54	1.31	1.2	0.55	−0.50	71.2	31.62	−0.39
Morocco	88.5	0.62	2.59	9.9	0.07	−0.17	570.5	4.00	−0.06
Mozambique	69.4	0.80	0.83	43.3	0.51	0.55	2438.4	27.77	0.54
Myanmar	22.3	0.11	0.19	14.8	0.07	−0.49	808.9	3.88	−0.49
Namibia	20.4	2.33	1.74	9.3	1.08	0.67	511.0	58.55	0.68
Nauru	0.00335	0.09	0.41	0.0023	0.06	0.17	0.1	3.24	0.18
Nepal	22.9	0.20	1.96	10.0	0.09	0.46	560.9	4.98	0.49
Netherlands	784.8	14.45	2.24	44.0	0.80	−0.72	2736.1	50.11	−0.42
New Zealand	407.8	22.50	1.03	23.8	1.31	−0.82	1479.9	81.66	−0.64
Nicaragua	7.3	0.29	2.39	2.2	0.09	0.48	122.3	4.80	0.52
Niger	5.5	0.09	0.03	3.2	0.06	−0.62	180.8	3.06	−0.63
Nigeria	316.3	0.48	1.25	155.5	0.24	0.23	8654.0	13.16	0.25
Niue	0.000619	0.11	0.54	0.000329	0.06	−0.16	0.0185	3.40	−0.12
North Macedonia	49.4	5.55	1.86	8.8	0.97	−0.95	489.9	54.67	−0.84
Northern Mariana Islands	0.00871	0.05	0.88	0.00433	0.02	0.63	0.2	1.34	0.66
Norway	269.9	14.22	1.80	17.6	0.91	−0.60	1058.9	55.48	−0.36
Oman	6.2	0.24	3.06	0.4	0.02	−0.24	25.8	0.98	0.08
Pakistan	357.1	0.42	1.38	178.9	0.21	0.53	10135.8	12.00	0.58
Palau	0.0322	0.51	0.56	0.0167	0.26	−0.03	0.9	15.03	0.04
Palestine	6.9	0.39	1.67	0.5	0.03	−0.99	33.5	1.91	−0.78
Panama	14.3	0.93	3.61	3.1	0.20	1.74	174.8	11.38	1.82
Papua New Guinea	1.9	0.05	0.32	1.4	0.04	0.24	80.4	2.16	0.26
Paraguay	23.0	0.83	1.65	7.1	0.26	0.33	394.1	14.32	0.35
Peru	78.6	0.56	2.25	18.3	0.13	−0.87	1011.6	7.20	−0.79
Philippines	59.6	0.14	−0.07	37.3	0.09	−0.30	2047.7	4.87	−0.31
Poland	642.0	4.36	2.12	119.4	0.78	−0.96	6615.6	43.95	−0.80
Portugal	176.0	5.02	2.52	16.0	0.44	−1.08	925.4	26.10	−0.92
Puerto Rico	16.2	1.51	2.04	2.4	0.22	−0.50	138.2	12.73	−0.38
Qatar	10.7	0.52	−0.02	0.5	0.03	−3.66	33.3	1.63	−3.26
Republic of Korea	282.5	1.50	4.61	16.4	0.08	−1.31	1018.7	5.33	−0.88
Republic of Moldova	27.5	1.79	2.02	6.8	0.43	0.66	366.5	23.73	0.68
Romania	206.9	3.17	2.61	35.0	0.52	−0.47	1934.2	29.19	−0.34
Russian Federation	2784.6	4.91	2.53	407.4	0.70	0.00299	22757.2	39.60	0.10
Rwanda	37.7	0.82	−0.82	20.1	0.44	−1.96	1126.9	24.36	−1.95
Saint Kitts and Nevis	0.0376	0.16	−1.29	0.0128	0.05	−3.01	0.7	2.87	−2.98
Saint Lucia	0.5	0.79	0.87	0.2	0.24	−0.55	9.3	13.37	−0.50
Saint Vincent and the Grenadines	0.4	1.01	1.34	0.1	0.33	0.36	7.5	18.11	0.33
Samoa	0.3	0.47	0.43	0.2	0.31	0.04	10.7	16.49	0.03
San Marino	1.1	11.27	1.58	0.061	0.60	−0.01	3.8	38.06	0.19
Sao Tome and Principe	0.0121	0.02	1.53	0.00494	0.0066	0.27	0.3	0.36	0.32
Saudi Arabia	62.3	0.29	5.53	4.1	0.02	1.02	246.4	1.14	1.35
Senegal	7.5	0.15	0.62	3.7	0.08	−0.13	206.7	4.24	−0.12
Serbia	119.8	3.56	1.47	21.6	0.63	−1.19	1190.5	35.18	−1.10
Seychelles	0.2	0.46	2.58	0.096	0.22	1.65	5.1	12.17	1.66
Sierra Leone	3.8	0.13	1.09	2.0	0.07	0.42	110.4	3.90	0.44
Singapore	24.2	0.99	2.88	1.5	0.06	−1.39	94.1	3.77	−0.98
Slovakia	135.6	6.33	2.07	16.8	0.76	−0.74	948.2	43.84	−0.56
Slovenia	96.1	13.53	3.12	5.8	0.78	−0.95	355.6	49.02	−0.54
Solomon Islands	0.2	0.06	1.11	0.1	0.05	0.91	6.2	2.67	0.92
Somalia	31.1	0.50	−0.30	22.4	0.36	−0.43	1236.0	19.64	−0.42
South Africa	199.1	0.84	0.47	84.0	0.36	−0.33	4545.6	19.26	−0.36
South Sudan	15.3	0.56	0.45	9.0	0.33	−0.07	497.1	18.04	−0.05
Spain	1159.8	7.55	1.54	68.4	0.42	−1.73	4179.7	26.75	−1.42
Sri Lanka	12.1	0.15	2.74	4.6	0.06	0.82	251.4	3.08	0.83
Sudan	69.7	0.47	1.77	10.3	0.07	−0.75	600.5	4.05	−0.63
Suriname	0.7	0.36	0.88	0.3	0.15	−0.06	17.4	8.40	−0.05
Sweden	429.8	12.49	2.16	24.5	0.71	−0.26	1539.5	44.59	0.08
Switzerland	355.4	11.31	0.96	16.7	0.52	−1.48	1078.9	33.98	−1.20
Syrian Arab Republic	24.0	0.54	4.14	1.5	0.03	0.16	92.7	2.05	0.51
Taiwan (Province of China)	43.7	0.46	1.43	15.6	0.16	0.33	831.4	8.69	0.28
Tajikistan	10.7	0.29	−0.17	4.8	0.13	−0.68	268.4	7.30	−0.66
Thailand	42.6	0.18	0.92	16.0	0.06	−0.85	860.3	3.48	−0.85
Timor-Leste	0.3	0.08	1.20	0.2	0.05	0.65	12.3	2.93	0.66
Togo	4.5	0.16	0.72	2.3	0.08	0.0000644	124.3	4.50	0.00426
Tokelau	0.000451	0.10	0.79	0.000247	0.05	−0.05	0.0142	3.06	−0.00884
Tonga	0.0214	0.07	0.75	0.0125	0.04	0.39	0.7	2.11	0.41
Trinidad and Tobago	2.1	0.38	1.62	0.6	0.11	0.01	35.2	6.35	0.07
Tunisia	35.2	0.74	3.19	2.0	0.04	0.03	121.8	2.55	0.35
Turkey	793.9	2.39	3.12	48.0	0.14	−1.59	2958.2	8.89	−1.22
Turkmenistan	9.8	0.50	−1.32	3.5	0.18	−2.41	198.0	10.26	−2.39
Tuvalu	0.00287	0.07	0.40	0.00194	0.05	−0.06	0.1	2.51	−0.07
Uganda	190.7	1.55	1.74	99.9	0.83	1.00	5693.6	46.21	1.04
Ukraine	571.9	3.39	1.66	174.9	1.01	0.76	9500.8	55.66	0.74
United Arab Emirates	77.1	1.48	1.68	6.6	0.11	−1.17	379.4	6.85	−0.90
United Kingdom	2842.9	12.30	2.57	185.9	0.79	0.10	11374.1	48.92	0.34
United Republic of Tanzania	137.5	0.76	0.00311	71.6	0.40	−0.60	4034.9	22.16	−0.58
United States Virgin Islands	0.6	2.48	2.59	0.2	0.66	1.34	9.2	39.03	1.61
United States of America	11134.8	9.75	−1.28	535.8	0.46	−2.79	34134.6	29.75	−2.55
Uruguay	41.8	3.45	2.78	7.6	0.62	0.94	427.6	35.13	1.05
Uzbekistan	37.5	0.28	1.51	13.2	0.10	0.36	743.5	5.52	0.40
Vanuatu	0.0738	0.07	0.38	0.0551	0.05	0.27	3.1	2.90	0.28
Venezuela (Bolivarian Republic of)	66.5	0.67	3.51	20.2	0.20	1.72	1114.4	11.18	1.86
Viet Nam	63.8	0.16	2.07	27.9	0.07	0.57	1507.8	3.70	0.57
Yemen	33.2	0.30	1.98	6.7	0.06	−0.01	378.0	3.36	0.10
Zambia	91.7	1.47	2.71	49.7	0.81	1.99	2801.3	44.87	1.97
Zimbabwe	77.2	1.52	2.79	45.3	0.90	3.20	2489.1	49.00	3.23

Abbreviations: ASDR, age-standardized death rate; ASIR, age-standardized incidence rate; DALY, disability-adjusted life year; EAPC, estimated annual percentage change.

#### Nonmelanoma skin cancer

The global burden of BCC increased substantially. Incident cases more than doubled, from 122,043 in 1990 to 258,700 in 2021, with the ASIR rising significantly (EAPC = 0.99%; 95% CI = 0.71%–1.27%) (Table 1). This trend was consistent across all SDI quintiles. In 2021, high-SDI regions carried the heaviest burden; the United States reported the most incident cases, and China had the fastest-rising ASIR (Figure 1 and Tables 4 and 5).

**Table 4:** Incidence, Prevalence, DALY Numbers, and ASRs in 2021, and EAPCs of Basal Cell Carcinoma for Different SDI and GBD Regions

Location	Incidence			Prevalence			DALYs		
	Case Number (95% UI)	ASR (per 100,000) (95% UI)	1990–2021 EAPC (95% CI)	Case Number (95% UI)	ASR (per 100,000) (95% UI)	1990–2021 EAPC (95% CI)	Case Number (95% UI)	ASR (per 100,000) (95% UI)	1990–2021 EAPC (95% CI)
SDI									
High SDI	157,230 (124,770–195,897)	23.03 (15.04–33.05)	2.22 (1.76–2.68)	18,374 (13,451–25,678)	4.51 (3.29–6.33)	1.88 (1.49–2.27)	79 (34–158)	0.02 (0.008–0.039)	1.8 (1.42–2.17)
High-middle SDI	37,381 (23,578–55,287)	3.44 (2.33–4.9)	1.09 (0.68–1.49)	6153 (3553–9997)	1.22 (0.7–1.99)	1.1 (0.74–1.45)	27 (10–56)	0.005 (0.002–0.011)	1.1 (0.74–1.45)
Middle SDI	48,281 (30,115–72,505)	3.28 (2.18–4.71)	0.85 (0.65–1.05)	6955 (3967–11,420)	0.74 (0.42–1.21)	0.83 (0.64–1.03)	31 (11–63)	0.003 (0.001–0.007)	0.84 (0.64–1.03)
Low-middle SDI	11,740 (6706–18,944)	1.47 (0.88–2.29)	0.62 (0.54–0.7)	1548 (822–2663)	0.22 (0.12–0.38)	0.59 (0.51–0.67)	7 (2–15)	0.001 (0–0.002)	0.59 (0.51–0.66)
Low SDI	4004 (1996–6929)	1.1 (0.57–1.88)	0.14 (0.11–0.17)	500 (231–920)	0.14 (0.07–0.26)	0.13 (0.1–0.16)	2 (1–5)	0.001 (0–0.001)	0.13 (0.1–0.16)
GBD Regions									
High-income Asia Pacific	601 (297–1034)	0.84 (0.43–1.43)	0.61 (0.58–0.65)	125 (55–237)	0.21 (0.09–0.4)	0.57 (0.54–0.6)	1 (0–1)	0.001 (0–0.002)	0.57 (0.53–0.6)
High-income North America	142,208 (114,215–175,136)	60.32 (38.66–87.53)	2.82 (2.25–3.39)	15,605 (11,706–21,407)	11.99 (8.98–16.48)	2.55 (2.03–3.07)	67 (29–134)	0.052 (0.022–0.103)	2.45 (1.95–2.96)
Western Europe	13,205 (8089–19,716)	9.24 (6.86–12.18)	−0.05 (−0.24 to 0.14)	2430 (1373–3984)	1.64 (0.92–2.7)	−0.01 (−0.18 to 0.17)	11 (4–22)	0.007 (0.003–0.015)	−0.01 (−0.18 to 0.17)
Australasia	859 (522–1289)	8.3 (5–12.2)	−0.17 (−0.23 to −0.11)	152 (83–251)	1.35 (0.74–2.23)	−0.17 (−0.22 to -0.13)	1 (0–1)	0.006 (0.002–0.012)	−0.18 (−0.22 to −0.14)
Andean Latin America	672 (373–1063)	3.14 (2.24–4.28)	−0.62 (−0.77 to −0.48)	105 (52–184)	0.42 (0.21–0.73)	−0.59 (−0.72 to −0.47)	0 (0–1)	0.002 (0.001–0.004)	−0.6 (−0.73 to −0.47)
Tropical Latin America	13,596 (9062–19,261)	16.91 (12.78–22.05)	0.18 (0.03–0.33)	1747 (1092–2707)	1.88 (1.17–2.92)	0.18 (0.06–0.31)	8 (3–16)	0.008 (0.003–0.017)	0.18 (0.06–0.31)
Central Latin America	7221 (4270–11,021)	7.5 (4.53–11.36)	0 (0–0.01)	1055 (578–1759)	1.1 (0.6–1.83)	−0.01 (−0.03 to 0)	5 (2–10)	0.005 (0.002–0.01)	−0.01 (−0.03 to 0.01)
Southern Latin America	1443 (849–2194)	5.82 (3.73–8.46)	−0.1 (−0.18 to −0.03)	238 (126–399)	0.92 (0.48–1.54)	−0.09 (−0.14 to −0.03)	1 (0–2)	0.004 (0.001–0.008)	−0.08 (−0.14 to −0.03)
Caribbean	272 (146–450)	2.06 (1.21–3.13)	−1 (−1.13 to −0.88)	43 (21–74)	0.25 (0.12–0.43)	−1.22 (−1.37 to −1.07)	0 (0–0)	0.001 (0–0.002)	−1.22 (−1.37 to −1.07)
Central Europe	2275 (1393–3423)	4.69 (3.33–6.49)	0.66 (0.52–0.8)	398 (221–657)	0.9 (0.49–1.49)	0.58 (0.45–0.71)	2 (1–4)	0.004 (0.001–0.008)	0.58 (0.45–0.7)
Eastern Europe	3177 (1833–4903)	3.21 (1.9–4.92)	0.52 (0.29–0.75)	435 (231–738)	0.53 (0.28–0.9)	0.48 (0.27–0.69)	2 (1–4)	0.002 (0.001–0.005)	0.48 (0.27–0.69)
Central Asia	1828 (1049–2815)	4.95 (2.93–7.54)	0 (−0.01 to 0.02)	258 (137–439)	0.7 (0.37–1.19)	−0.07 (−0.09 to −0.06)	1 (0–2)	0.003 (0.001–0.007)	−0.07 (−0.09 to −0.06)
North Africa and Middle East	4084 (2240–6702)	1.82 (1.07–2.8)	−0.46 (−0.69 to −0.24)	708 (350–1248)	0.28 (0.14–0.5)	−0.52 (−0.74 to −0.29)	3 (1–7)	0.001 (0–0.003)	−0.51 (−0.74 to −0.29)
South Asia	5651 (2708–10,088)	0.65 (0.29–1.19)	0.69 (0.64–0.75)	748 (328–1416)	0.1 (0.05–0.2)	0.71 (0.65 to 0.76)	3 (1–8)	0 (0–0.001)	0.7 (0.65–0.76)
Southeast Asia	1568 (629–2981)	0.66 (0.36–1.11)	−0.47 (−0.59 to −0.35)	238 (87–486)	0.09 (0.03–0.18)	−0.49 (−0.6 to −0.37)	1 (0–2)	0 (0–0.001)	−0.49 (−0.6 to −0.37)
East Asia	53,711 (33,518–79,841)	1.85 (1.06–2.89)	3.34 (2.61–4.08)	8476 (4870–13,757)	1.57 (0.89–2.55)	3.24 (2.57 to 3.91)	37 (14–77)	0.007 (0.003–0.014)	3.24 (2.57–3.91)
Oceania	12 (2–28)	0.23 (0.04–0.54)	0.04 (0.02–0.06)	2 (0–4)	0.03 (0.01–0.08)	−0.01 (−0.03 to 0)	0 (0–0)	0 (0–0)	−0.01 (−0.03 to 0.01)
Western Sub-Saharan Africa	1893 (915–3303)	1.32 (0.66–2.25)	0 (−0.01 to 0.01)	243 (111–450)	0.17 (0.08–0.31)	−0.05 (−0.07 to −0.03)	1 (0–2)	0.001 (0–0.002)	−0.05 (−0.07 to −0.03)
Eastern Sub-Saharan Africa	1847 (948–3124)	1.42 (0.76–2.34)	−0.07 (−0.09 to −0.05)	224 (108–403)	0.17 (0.08–0.3)	−0.09 (−0.1 to −0.07)	1 (0–2)	0.001 (0–0.002)	−0.09 (−0.1 to −0.07)
Central Sub-Saharan Africa	609 (302–1039)	1.47 (0.75–2.51)	0.02 (0.01–0.03)	77 (35–142)	0.19 (0.09–0.34)	0 (−0.01 to 0.01)	0 (0–1)	0.001 (0–0.002)	0 (−0.01 to 0.01)
Southern Sub-Saharan Africa	1969 (1131–3043)	5.47 (3.15–8.49)	0.34 (0.22–0.46)	234 (127–387)	0.74 (0.41–1.23)	0.36 (0.25–0.47)	1 (0–2)	0.003 (0.001–0.007)	0.36 (0.24–0.47)

Abbreviations: ASR, age-standardized rate; CI, confidence interval; DALY, disability-adjusted life year; EAPC, estimated annual percentage change; GBD, Global Burden of Disease; SDI, Sociodemographic Index; UI, uncertainty interval.

**Table 5:** Incidence, DALY Numbers, and Age-Standardized Rates of Basal Cell Carcinoma, across 204 Countries and Territories in 2021, Including EAPCs from 1990 to 2021

Location	Incidence			Disability-Adjusted Life Year		
	Number	ASIR	EAPC	Number	Age-Standardized DALY Rate	EAPC
Afghanistan	97.2	1.19	0.14	0.0495	0.000605	0.17
Albania	47.6	5.27	0.00985	0.0408	0.00452	−0.03
Algeria	266.6	1.51	−0.02	0.2	0.00106	−0.04
American Samoa	0.0367	0.23	−0.00063	0.0000249	0.000158	−0.00236
Andorra	2.9	8.34	0.0013	0.00246	0.00723	0.00732
Angola	135.7	1.48	−0.00921	0.0752	0.000822	−0.02
Antigua and Barbuda	0.4	1.09	0.05	0.0003	0.000844	−0.01
Argentina	1007.9	5.70	−0.25	0.7	0.004	−0.20
Armenia	59.7	4.92	0.02	0.0477	0.00393	−0.01
Australia	713.9	7.52	−0.21	0.6	0.00594	−0.21
Austria	213.9	6.71	0.69	0.2	0.00578	0.64
Azerbaijan	215.8	4.91	0.000658	0.2	0.00341	−0.03
Bahamas	1.7	1.10	0.05	0.000946	0.00062	0.05
Bahrain	5.9	0.75	0.02	0.00515	0.000659	−0.03
Bangladesh	86.1	0.13	−0.00676	0.0593	0.0000931	−0.03
Barbados	1.1	1.09	0.04	0.000822	0.000782	0.01
Belarus	156.0	4.25	0.42	0.1	0.00284	0.40
Belgium	298.9	7.62	−0.03	0.2	0.00612	−0.07
Belize	1.8	1.10	−0.06	0.00108	0.000681	−0.07
Benin	53.2	1.37	0.03	0.0311	0.000807	−0.00238
Bermuda	0.1	0.49	−0.11	0.0000925	0.000422	−0.14
Bhutan	0.4	0.13	−0.00637	0.000304	0.0000923	−0.03
Bolivia (Plurinational State of)	125.3	2.84	0.05	0.079	0.0018	0.01
Bosnia and Herzegovina	65.0	5.27	−0.00413	0.0568	0.00465	−0.05
Botswana	47.7	4.68	0.03	0.0236	0.00232	0.02
Brazil	13592.1	15.09	0.20	7.7	0.00855	0.20
Brunei Darussalam	2.4	1.15	−0.01	0.00166	0.000783	−0.07
Bulgaria	234.0	9.40	1.05	0.2	0.00644	0.93
Burkina Faso	89.0	1.37	0.04	0.0471	0.000729	0.03
Burundi	53.4	1.30	0.01	0.0276	0.000676	0.000537
Cabo Verde	3.2	1.38	0.10	0.00217	0.000939	−0.02
Cambodia	22.4	0.34	0.02	0.0129	0.000196	−0.01
Cameroon	136.2	1.38	0.04	0.07	0.000709	0.01
Canada	948.9	7.07	0.82	0.7	0.00549	0.73
Central African Republic	25.0	1.48	0.01	0.0117	0.000697	−0.0049
Chad	59.4	1.36	0.02	0.0321	0.000741	−0.00689
Chile	353.7	4.98	0.19	0.3	0.00397	0.16
China	53677.9	10.31	3.39	37.3	0.00715	3.30
Colombia	1367.3	7.21	0.00802	0.9	0.00485	0.02
Comoros	3.5	1.31	0.02	0.00232	0.000866	−0.03
Congo	28.9	1.48	0.02	0.0149	0.000765	−0.00564
Cook Islands	0.0128	0.23	0.00398	0.0000101	0.000181	0.06
Costa Rica	163.9	8.61	−0.38	0.1	0.00658	−0.31
Côte d’Ivoire	75.5	0.82	0.14	0.0382	0.000415	0.10
Croatia	62.7	4.12	0.11	0.0542	0.00359	0.02
Cuba	117.0	3.20	−0.72	0.094	0.00257	−0.70
Cyprus	34.8	5.63	0.26	0.0312	0.00507	0.26
Czechia	336.2	8.41	0.76	0.3	0.00701	0.66
Democratic People's Republic of Korea	21.1	0.21	−0.00738	0.0154	0.000151	−0.06
Democratic Republic of the Congo	402.4	1.48	0.03	0.2	0.000834	0.00954
Denmark	184.3	9.75	−1.68	0.1	0.00794	−1.42
Djibouti	6.6	1.30	0.0052	0.00389	0.000765	−0.04
Dominica	0.3	1.12	−0.00748	0.000173	0.000723	−0.02
Dominican Republic	46.9	1.12	0.13	0.0296	0.000708	0.10
Ecuador	238.8	3.60	−1.49	0.2	0.00244	−1.40
Egypt	426.1	1.12	−0.05	0.3	0.000758	−0.03
El Salvador	169.4	7.47	0.06	0.1	0.00445	0.08
Equatorial Guinea	7.5	1.50	0.07	0.00401	0.000797	0.07
Eritrea	29.9	1.30	0.01	0.0159	0.000693	−0.03
Estonia	20.2	4.22	−0.19	0.0147	0.00309	−0.22
Eswatini	19.3	4.68	0.05	0.00932	0.00227	0.03
Ethiopia	511.1	1.46	0.00526	0.3	0.000822	−0.02
Fiji	0.8	0.23	−0.00638	0.000518	0.000153	−0.02
Finland	157.3	8.44	−0.05	0.1	0.00657	−0.03
France	2189.6	10.23	−1.09	1.7	0.00792	−0.94
Gabon	9.0	1.48	−0.04	0.00507	0.00083	−0.05
Gambia	9.6	1.29	0.16	0.00538	0.000727	0.16
Georgia	64.5	4.95	0.01	0.045	0.00347	−0.05
Germany	1935.5	6.70	1.63	1.6	0.00566	1.46
Ghana	161.3	1.37	−0.01	0.0886	0.000753	−0.04
Greece	305.2	8.43	0.00674	0.3	0.00716	−0.007
Greenland	3.3	16.53	0.28	0.00165	0.00829	0.26
Grenada	0.4	1.12	0.08	0.000265	0.000726	0.05
Guam	0.1	0.23	0.00116	0.0000841	0.00016	0.02
Guatemala	414.7	7.48	0.03	0.2	0.00392	0.04
Guinea	46.8	1.22	−0.69	0.0257	0.000671	−0.71
Guinea-Bissau	8.8	1.36	0.00228	0.00437	0.000678	−0.02
Guyana	3.0	1.10	−0.01	0.00154	0.000567	−0.02
Haiti	52.7	1.09	0.02	0.0264	0.000545	0.01
Honduras	265.4	7.47	0.05	0.2	0.00422	0.04
Hungary	203.3	5.58	−0.25	0.1	0.00406	−0.24
Iceland	10.5	8.34	−0.03	0.00924	0.00732	−0.04
India	4597.2	0.83	0.64	2.7	0.000483	0.67
Indonesia	520.3	0.46	0.02	0.3	0.000298	−0.02
Iran (Islamic Republic of)	957.5	2.41	−0.73	0.7	0.00181	−0.70
Iraq	164.8	1.10	0.16	0.1	0.000733	0.15
Ireland	233.3	12.51	−1.19	0.2	0.01	−0.97
Israel	278.4	8.48	0.00895	0.2	0.00747	−0.05
Italy	2027.8	10.46	0.47	1.7	0.00864	0.37
Jamaica	15.5	1.43	−0.65	0.0116	0.00107	−0.61
Japan	357.7	0.94	0.46	0.3	0.000921	0.43
Jordan	78.9	1.67	−0.17	0.0665	0.00141	−0.18
Kazakhstan	365.4	5.00	0.02	0.2	0.0029	−0.01
Kenya	222.5	1.31	0.10	0.1	0.00071	0.08
Kiribati	0.1	0.23	−0.0009	0.0000559	0.000124	0.00223
Kuwait	28.2	1.03	0.59	0.0285	0.00103	0.64
Kyrgyzstan	120.2	4.98	−0.13	0.0743	0.00306	−0.11
Lao People's Democratic Republic	9.9	0.35	0.02	0.00561	0.000196	−0.00141
Latvia	24.0	3.69	0.82	0.0162	0.0025	0.76
Lebanon	64.4	2.73	−0.12	0.052	0.00219	−0.16
Lesotho	31.0	4.71	0.05	0.0146	0.00222	0.02
Liberia	25.8	1.38	0.02	0.0145	0.000779	−0.00077
Libya	23.6	0.74	−0.03	0.0169	0.000526	−0.04
Lithuania	42.8	4.75	−0.08	0.0291	0.00324	−0.11
Luxembourg	21.9	8.38	−0.00602	0.0177	0.00681	−0.04
Madagascar	116.2	1.29	−0.02	0.0688	0.000771	−0.02
Malawi	57.0	0.96	−0.78	0.0279	0.00047	−0.81
Malaysia	122.4	0.93	−0.02	0.0955	0.000722	−0.02
Maldives	1.0	0.36	0.15	0.000934	0.00032	0.20
Mali	131.1	2.11	−0.05	0.0729	0.00118	−0.06
Malta	14.9	9.17	−0.57	0.0134	0.00826	−0.54
Marshall Islands	0.0499	0.23	−0.00196	0.0000278	0.000127	−0.0011
Mauritania	17.7	1.37	−0.01	0.0113	0.000883	−0.05
Mauritius	1.7	0.34	0.02	0.00123	0.000255	0.02
Mexico	3840.2	7.76	0.02	2.5	0.00498	0.03
Micronesia (Federated States of)	0.0852	0.23	−0.00245	0.0000473	0.000127	−0.01
Monaco	0.9	8.50	−0.01	0.00073	0.00666	0.01
Mongolia	66.0	5.03	0.02	0.0357	0.00272	−0.00361
Montenegro	12.4	5.34	0.00984	0.00981	0.00425	−0.03
Morocco	93.9	0.66	−0.02	0.0644	0.000451	−0.04
Mozambique	113.2	1.30	0.01	0.0549	0.000634	−0.00467
Myanmar	72.1	0.35	−0.00018	0.0394	0.000189	−0.00927
Namibia	43.3	4.99	0.54	0.0239	0.00275	0.49
Nauru	0.00945	0.23	−0.00042	0.0000051	0.000125	−0.00031
Nepal	16.2	0.13	0.00153	0.0103	0.0000864	−0.03
Netherlands	298.7	5.42	0.46	0.3	0.00493	0.40
New Zealand	144.9	8.01	0.04	0.1	0.00582	−0.01
Nicaragua	183.4	7.22	0.02	0.1	0.00502	0.02
Niger	80.9	1.36	−0.02	0.0466	0.000792	−0.04
Nigeria	785.4	1.18	0.03	0.5	0.000692	−0.03
Niue	0.00123	0.23	0.00243	0.000000845	0.000159	0.01
North Macedonia	48.4	5.27	−0.03	0.0399	0.00438	−0.07
Northern Mariana Islands	0.0366	0.23	−0.00596	0.0000286	0.000178	−0.02
Norway	170.3	8.91	−0.03	0.1	0.00721	−0.02
Oman	36.5	1.42	−0.48	0.0262	0.00101	−0.48
Pakistan	950.9	1.09	0.76	0.5	0.000614	0.74
Palau	0.0145	0.23	−0.01	0.00000891	0.000141	−0.06
Palestine	20.1	1.17	0.02	0.0175	0.00102	0.00853
Panama	70.9	4.61	−0.41	0.0514	0.00334	−0.46
Papua New Guinea	8.9	0.23	0.06	0.00545	0.000142	0.05
Paraguay	3.9	0.14	−0.04	0.003	0.000106	−0.06
Peru	308.1	2.19	−0.05	0.2	0.00158	−0.08
Philippines	327.7	0.77	−0.11	0.2	0.000491	−0.13
Poland	613.3	3.89	1.34	0.5	0.00303	1.20
Portugal	171.0	4.57	1.27	0.1	0.00364	1.17
Puerto Rico	12.1	1.10	0.04	0.00858	0.000777	0.01
Qatar	14.4	0.70	0.02	0.0122	0.000592	−0.05
Republic of Korea	195.8	1.02	0.75	0.2	0.000831	0.76
Republic of Moldova	62.4	4.01	−0.01	0.0412	0.00266	−0.06
Romania	261.3	3.74	0.23	0.2	0.00274	0.18
Russian Federation	2106.3	3.62	0.83	1.2	0.00214	0.83
Rwanda	59.2	1.29	0.00299	0.0323	0.000708	−0.02
Saint Kitts and Nevis	0.3	1.12	0.05	0.000167	0.000689	0.07
Saint Lucia	0.8	1.12	0.08	0.000519	0.000738	0.04
Saint Vincent and the Grenadines	0.5	1.11	0.03	0.000281	0.000677	0.02
Samoa	0.2	0.23	−0.18	0.000103	0.00015	−0.20
San Marino	0.9	8.41	−0.00071	0.000771	0.00752	0.02
Sao Tome and Principe	1.0	1.38	0.04	0.000713	0.000951	0.00496
Saudi Arabia	184.2	0.87	−0.34	0.1	0.000607	−0.37
Senegal	131.7	2.70	0.00672	0.0797	0.00164	−0.02
Serbia	164.2	4.76	0.35	0.1	0.00377	0.28
Seychelles	0.1	0.35	0.24	0.000102	0.000244	0.18
Sierra Leone	38.8	1.37	0.02	0.0213	0.00076	−0.03
Singapore	44.8	1.66	0.41	0.044	0.00165	0.40
Slovakia	134.4	5.97	−0.67	0.1	0.00482	−0.56
Slovenia	58.9	7.59	1.99	0.0497	0.00647	1.75
Solomon Islands	0.5	0.23	0.000754	0.000277	0.000115	0.00109
Somalia	81.8	1.30	−0.00088	0.0431	0.000689	−0.02
South Africa	1662.7	7.08	0.44	0.9	0.00373	0.46
South Sudan	35.5	1.30	−0.00816	0.0199	0.000731	−0.02
Spain	1615.3	9.63	−1.50	1.3	0.0081	−1.33
Sri Lanka	24.1	0.30	−0.42	0.0177	0.000219	−0.40
Sudan	169.5	1.17	−0.00332	0.1	0.000694	−0.02
Suriname	2.3	1.10	0.02	0.00138	0.000664	0.00591
Sweden	176.8	5.05	−0.84	0.2	0.00429	−0.82
Switzerland	389.7	11.86	−1.49	0.3	0.00995	−1.27
Syrian Arab Republic	54.4	1.15	−0.05	0.0415	0.00088	−0.07
Taiwan (Province of China)	12.4	0.15	−7.03	0.0114	0.000133	−6.87
Tajikistan	174.0	4.96	−0.00294	0.1	0.00315	−0.03
Thailand	266.5	1.10	−0.77	0.2	0.000714	−0.79
Timor-Leste	1.5	0.34	−0.00331	0.000944	0.000222	−0.02
Togo	37.6	1.36	0.03	0.0201	0.00073	0.02
Tokelau	0.00105	0.23	−0.00258	0.000000674	0.000148	−0.00126
Tonga	0.0768	0.23	−0.05	0.0000592	0.000177	−0.06
Trinidad and Tobago	5.8	1.04	−0.18	0.00372	0.000664	−0.22
Tunisia	76.5	1.59	−0.64	0.0629	0.0013	−0.61
Turkey	1094.3	3.27	0.05	1.0	0.00293	0.06
Turkmenistan	94.5	4.97	0.00487	0.0559	0.00294	−0.04
Tuvalu	0.0103	0.23	−0.01	0.00000603	0.000136	−0.02
Uganda	242.1	2.01	−0.46	0.1	0.001	−0.47
Ukraine	765.6	4.37	−0.00249	0.5	0.00268	−0.06
United Arab Emirates	91.4	1.24	0.05	0.0736	0.000988	0.03
United Kingdom	2460.6	10.46	0.29	1.9	0.00814	0.31
United Republic of Tanzania	232.1	1.29	−0.01	0.1	0.000688	−0.03
United States Virgin Islands	0.3	1.11	0.10	0.000192	0.000745	0.07
United States of America	141253.5	120.73	2.84	66.4	0.06	2.48
Uruguay	81.1	6.57	0.20	0.0575	0.00466	0.18
Uzbekistan	668.2	4.99	0.00422	0.4	0.00301	−0.03
Vanuatu	0.3	0.23	−0.00026	0.000145	0.000132	0.000436
Venezuela (Bolivarian Republic of)	745.5	7.24	−0.01	0.5	0.00472	−0.02
Viet Nam	195.7	0.49	−1.10	0.2	0.000381	−1.07
Yemen	131.7	1.17	0.02	0.077	0.000686	0.00427
Zambia	80.8	1.31	0.02	0.039	0.000633	0.02
Zimbabwe	165.4	3.29	−0.49	0.0816	0.00163	−0.49

Abbreviations: ASIR, age-standardized incidence rate; DALY, disability-adjusted life year; EAPC, estimated annual percentage change.

The global number of SCC incident cases increased from 19,975 in 1990 to 33,352 in 2021, whereas the ASIR remained relatively stable (EAPC = 0.25%; 95% CI = −0.07% to 0.57%). The global age-standardized death rate for SCC decreased from 0.10 per 100,000 population in 1990 to 0.08 in 2021 (EAPC = −0.37%; 95% CI = −0.46% to −0.28%). Cases were consistently higher in men, who also had a greater DALY and mortality burden. Although mortality and DALY rates declined overall, this improvement was not observed in low- and low-middle-SDI regions. Similar to BCC, high-SDI regions had the highest burden in 2021, with the United States reporting the most cases and China having the fastest-growing ASIR. National-level patterns were generally consistent with regional trends (Figure 1, Figure 2, Figure 3 and Tables 6 and 7).

**Table 6:** Incidence, Prevalence, DALY, Death Numbers, ASRs in 2021, and EAPCs of Squamous Cell Carcinoma for Different SDI and GBD Regions

Location	Incidence			Prevalence			DALYs			Deaths		
	Case Number (95% UI)	ASR (per 100,000) (95% UI)	1990–2021 EAPC (95% CI)	Case Number (95% UI)	ASR (per 100,000) (95% UI)	1990–2021 EAPC (95% CI)	Case Number (95% UI)	ASR (per 100,000) (95% UI)	1990–2021 EAPC (95% CI)	Case Number (95% UI)	ASR (per 100,000) (95% UI)	1990–2021 EAPC (95% CI)
SDI												
High SDI	24,876 (19,061–32,603)	6.02 (4.6–7.92)	1.27 (0.84–1.69)	26,937 (19,249–38,616)	6.55 (4.66–9.41)	1.28 (0.85–1.72)	13,164 (12,334–14,448)	3.33 (3.12–3.65)	−1.24 (−1.55 to −0.93)	223 (213–244)	0.06 (0.05–0.06)	−1.45 (−1.76 to −1.13)
High-middle SDI	3381 (1690–5989)	0.67 (0.33–1.19)	1.2 (0.78–1.62)	6012 (2879–11,095)	1.25 (0.59–2.31)	0.86 (0.49–1.23)	25,352 (22,186–30,258)	5.11 (4.47–6.11)	−0.64 (−0.79 to −0.49)	472 (413–564)	0.09 (0.08–0.11)	−0.66 (−0.81 to −0.5)
Middle SDI	4210 (2084–7571)	0.45 (0.22–0.81)	0.65 (0.39–0.91)	7346 (3549–13,490)	0.8 (0.38–1.46)	0.14 (−0.12 to 0.4)	54,820 (42,585–63,406)	5.86 (4.55–6.78)	−0.19 (−0.32 to −0.06)	1013 (785–1171)	0.11 (0.08–0.12)	−0.2 (−0.33 to -0.06)
Low-middle SDI	632 (275–1209)	0.09 (0.04–0.17)	0.08 (−0.12 to 0.27)	987 (411–2012)	0.14 (0.06–0.28)	−0.01 (−0.17 to 0.15)	27,257 (22,037–31,793)	3.84 (3.11–4.48)	0.79 (0.68–0.9)	501 (406–584)	0.07 (0.06–0.08)	0.8 (0.69–0.9)
Low SDI	248 (110–469)	0.07 (0.03–0.13)	0.21 (0.1–0.33)	383 (158–797)	0.1 (0.04–0.21)	0.09 (0–0.17)	10,095 (4616–14,225)	2.92 (1.34–4.11)	1.04 (0.99–1.1)	182 (84–256)	0.05 (0.02–0.08)	1 (0.95–1.06)
GBD regions												
High-income Asia Pacific	53 (21–104)	0.09 (0.03–0.17)	0.66 (0.62–0.71)	88 (32–188)	0.15 (0.05–0.32)	0.56 (0.54–0.57)	1039 (919–1340)	1.68 (1.48–2.17)	−1.77 (−1.88 to −1.66)	20 (18–25)	0.03 (0.03–0.04)	−1.73 (−1.85 to −1.61)
High-income North America	22,600 (17,643–29,011)	17.24 (13.43–22.16)	1.73 (1.2–2.26)	23,970 (17,448–33,952)	18.31 (13.3–25.97)	1.73 (1.18–2.28)	6428 (5865–7212)	5.03 (4.59–5.63)	−1.01 (−1.62 to −0.4)	99 (93–103)	0.08 (0.07–0.08)	−1.48 (−2.13 to −0.82)
Western Europe	1369 (757–2265)	0.9 (0.49–1.5)	0.47 (0.39–0.55)	1722 (914–2965)	1.14 (0.6–1.97)	0.4 (0.33–0.47)	4416 (4213–4639)	3.02 (2.89–3.18)	−0.97 (−1.16 to −0.78)	82 (78–86)	0.06 (0.05–0.06)	−0.97 (−1.16 to −0.78)
Australasia	843 (488–1373)	7.45 (4.31–12.14)	−1.11 (−1.28 to −0.94)	875 (498–1420)	7.76 (4.41–12.61)	−0.82 (−0.92 to −0.71)	719 (621–836)	6.41 (5.53–7.45)	−1.06 (−1.46 to −0.65)	13 (11–15)	0.11 (0.1–0.13)	−1 (−1.44 to −0.57)
Andean Latin America	16 (7–32)	0.06 (0.03–0.12)	−0.79 (−0.91 to −0.67)	29 (11–61)	0.11 (0.04–0.24)	−0.7 (−0.84 to −0.55)	1708 (1280–2218)	6.74 (5.05–8.76)	1.56 (1.23–1.89)	31 (23–41)	0.12 (0.09–0.16)	1.55 (1.22–1.87)
Tropical Latin America	697 (378–1189)	0.75 (0.41–1.28)	0.25 (0.03–0.47)	873 (451–1542)	0.95 (0.49–1.68)	0.24 (0.05–0.44)	7734 (7354–8129)	8.36 (7.95–8.79)	−0.04 (−0.19 to 0.11)	144 (137–152)	0.15 (0.15–0.16)	−0.09 (−0.25 to 0.06)
Central Latin America	271 (133–486)	0.28 (0.14–0.51)	−0.04 (−0.05 to −0.03)	452 (203–857)	0.47 (0.21–0.89)	−0.11 (−0.12 to −0.09)	6087 (5348–6891)	6.33 (5.56–7.17)	−1.21 (−1.35 to −1.07)	112 (98–127)	0.12 (0.1–0.13)	−1.23 (−1.37 to −1.09)
Southern Latin America	91 (46–158)	0.35 (0.18–0.61)	0 (−0.04 to 0.05)	134 (65–245)	0.52 (0.25–0.94)	−0.12 (−0.15 to −0.09)	1125 (1015–1239)	4.35 (3.92–4.79)	0.47 (0.19–0.76)	21 (19–23)	0.08 (0.07–0.09)	0.44 (0.15–0.73)
Caribbean	25 (13–43)	0.14 (0.07–0.25)	−1.55 (−1.68 to −1.42)	37 (18–68)	0.22 (0.1–0.4)	−1.47 (−1.66 to −1.28)	1457 (1119–1807)	8.47 (6.51–10.51)	1.43 (1.11–1.75)	27 (21–34)	0.16 (0.12–0.2)	1.44 (1.12–1.76)
Central Europe	148 (78–251)	0.32 (0.17–0.56)	0.34 (0.28–0.4)	226 (111–407)	0.51 (0.25–0.94)	0.21 (0.15–0.28)	915 (805–1046)	2.09 (1.84–2.38)	−3.99 (−4.31 to −3.67)	17 (15–20)	0.04 (0.03–0.04)	−4.01 (−4.33 to −3.68)
Eastern Europe	266 (135–470)	0.33 (0.16–0.58)	0.29 (0.23–0.35)	402 (189–765)	0.51 (0.24–0.98)	0.21 (0.19–0.23)	4866 (4336–5411)	5.91 (5.27–6.58)	−1.16 (−1.67 to −0.64)	93 (83–103)	0.11 (0.1 –0.12)	−1.14 (−1.64 to −0.63)
Central Asia	13 (3–30)	0.04 (0.01–0.08)	−0.07 (−0.1 to −0.05)	24 (6–59)	0.07 (0.02–0.16)	−0.25 (−0.29 to −0.22)	2524 (2185–2874)	6.88 (5.96–7.83)	2.32 (2.11–2.53)	47 (41–53)	0.13 (0.11–0.15)	2.33 (2.12–2.54)
North Africa and Middle East	371 (184–672)	0.15 (0.07–0.27)	−0.34 (−0.39 to −0.29)	611 (279–1160)	0.24 (0.11–0.46)	−0.35 (−0.4 to −0.31)	3912 (3043–5194)	1.57 (1.22–2.09)	−0.09 (−0.33 to 0.16)	71 (55–94)	0.03 (0.02–0.04)	−0.09 (−0.33 to 0.16)
South Asia	475 (164–982)	0.07 (0.02–0.14)	−0.18 (−0.44 to 0.07)	726 (235–1643)	0.1 (0.03–0.23)	−0.22 (−0.5 to 0.06)	19,422 (16,021–25,428)	2.74 (2.26–3.59)	0.45 (0.35–0.54)	360 (297–472)	0.05 (0.04–0.07)	0.46 (0.37–0.55)
Southeast Asia	221 (96–423)	0.08 (0.04–0.16)	0.32 (0.23–0.4)	389 (159–793)	0.14 (0.06–0.29)	0.27 (0.22–0.31)	18,541 (13,255–23,033)	6.78 (4.84–8.42)	0.05 (−0.09 to 0.19)	341 (244–424)	0.12 (0.09–0.15)	0.07 (−0.07 to 0.2)
East Asia	5054 (2377–9366)	0.94 (0.44–1.75)	2.21 (1.52–2.9)	9925 (4542–18,685)	1.98 (0.89–3.72)	1.34 (0.83–1.85)	37,300 (28,355–46,551)	7.05 (5.36–8.79)	0.29 (0.11–0.47)	690 (522–864)	0.13 (0.1–0.16)	0.27 (0.09–0.45)
Oceania	1 (0–3)	0.03 (0.01–0.06)	0.02 (0.01–0.03)	3 (1–8)	0.06 (0.01–0.16)	−0.09 (−0.18 to 0)	268 (152–430)	5.58 (3.17–8.91)	0.87 (0.82–0.91)	5 (3–8)	0.11 (0.06–0.17)	0.83 (0.79–0.88)
Western Sub-Saharan Africa	97 (45–180)	0.06 (0.03–0.11)	0.32 (0.23–0.4)	150 (67–305)	0.1 (0.04–0.19)	0.13 (0.1–0.17)	6110 (1552–9484)	4.15 (1.07–6.43)	2.48 (2.23–2.73)	107 (28–166)	0.07 (0.02–0.11)	2.43 (2.18–2.67)
Eastern Sub-Saharan Africa	108 (51–202)	0.08 (0.04–0.14)	0.47 (0.41–0.53)	167 (71–340)	0.12 (0.05–0.24)	0.3 (0.28–0.32)	3785 (1171–6004)	2.88 (0.88–4.57)	0.84 (0.81–0.87)	69 (21–109)	0.05 (0.02–0.08)	0.84 (0.8–0.87)
Central Sub-Saharan Africa	15 (4–35)	0.03 (0.01–0.08)	0.02 (0–0.04)	26 (6–63)	0.06 (0.01–0.14)	−0.18 (−0.22 to −0.15)	1205 (373–2020)	2.93 (0.9–4.91)	1.25 (1.11–1.39)	22 (7–37)	0.05 (0.02–0.09)	1.24 (1.1–1.38)
Southern Sub-Saharan Africa	616 (335–1039)	1.96 (1.07 –3.3)	0.34 (0.27–0.41)	843 (438–1446)	2.68 (1.39–4.58)	0.24 (0.18–0.3)	1240 (951–1539)	3.96 (3.03–4.92)	1.2 (0.76–1.65)	23 (17–28)	0.07 (0.06–0.09)	1.3 (0.86–1.75)

Abbreviations: ASR, age-standardized rate; CI, confidence interval; DALY, disability-adjusted life year; EAPC, estimated annual percentage change; GBD, Global Burden of Disease; SDI, Sociodemographic Index; UI, uncertainty interval.

**Table 7:** Incidence, Death, and DALY Numbers, and Age-Standardized Rates of Squamous Cell Carcinoma, across 204 Countries and Territories in 2021, Including EAPCs from 1990 to 2021

Location	Incidence			Death			Disability-Adjusted Life Year		
	Number	ASIR	EAPC	Number	ASDR	EAPC	Number	Age-Standardized DALY Rate	EAPC
Afghanistan	3.9	0.05	0.48	0.2	0.00284	3.95	14.4	0.16	3.82
Albania	3.0	0.33	0.08	0.6	0.07	−1.44	33.2	3.66	−1.52
Algeria	47.5	0.27	0.88	0.3	0.0019	3.54	21.9	0.13	2.92
American Samoa	0.00417	0.03	0.0000307	0.0286	0.16	2.06	1.5	8.55	2.15
Andorra	0.3	0.82	−0.000208	0.0165	0.05	−1.07	0.9	2.67	−1.04
Angola	3.4	0.03	−0.03	5.3	0.06	1.46	289.3	3.15	1.48
Antigua and Barbuda	0.00879	0.03	0.05	0.0318	0.09	7.74	1.7	4.83	7.73
Argentina	63.9	0.36	−0.03	12.9	0.07	−0.31	695.6	3.96	−0.27
Armenia	0.4	0.04	0.01	1.2	0.10	2.82	62.7	5.30	2.81
Australia	696.2	7.31	−1.30	12.0	0.13	−1.15	669.1	7.13	−1.20
Austria	15.9	0.49	0.51	1.7	0.05	−1.08	92.9	2.97	−1.05
Azerbaijan	1.6	0.04	−0.00775	2.7	0.06	−0.28	144.3	3.32	−0.29
Bahamas	0.0378	0.03	−0.00452	0.2	0.14	1.12	11.2	7.38	1.08
Bahrain	0.1	0.02	−0.02	0.2	0.03	1.97	13.3	1.76	1.99
Bangladesh	10.0	0.02	−0.00564	28.7	0.05	0.05	1558.9	2.51	0.08
Barbados	0.0257	0.03	0.05	0.0471	0.04	−0.20	2.5	2.41	−0.24
Belarus	12.9	0.35	0.30	3.3	0.09	−1.29	175.2	4.80	−1.34
Belgium	22.1	0.55	−0.09	2.2	0.06	−0.78	116.3	2.99	−0.80
Belize	0.0425	0.03	−0.03	0.2	0.15	1.49	12.7	7.93	1.48
Benin	1.0	0.02	0.02	2.8	0.07	2.27	159.9	3.97	2.33
Bermuda	0.00289	0.02	−0.02	0.0172	0.08	3.05	0.9	4.07	3.05
Bhutan	0.0518	0.02	0.02	0.1	0.04	0.46	7.4	2.31	0.47
Bolivia (Plurinational State of)	2.5	0.06	0.01	5.9	0.13	0.62	325.7	7.29	0.64
Bosnia and Herzegovina	4.2	0.33	0.03	0.4	0.03	−1.92	19.1	1.62	−1.90
Botswana	6.7	0.66	0.17	0.4	0.04	0.75	20.3	1.98	0.73
Brazil	697.0	0.77	0.27	141.3	0.16	−0.10	7568.9	8.43	−0.05
Brunei Darussalam	0.2	0.09	−0.00161	0.1	0.06	−0.98	6.7	3.14	−1.04
Bulgaria	9.7	0.38	−0.02	1.6	0.07	−1.66	85.5	3.49	−1.71
Burkina Faso	1.6	0.02	−0.000466	4.4	0.06	2.16	247.5	3.63	2.24
Burundi	1.4	0.03	0.08	2.0	0.05	0.37	108.8	2.67	0.38
Cabo Verde	0.0551	0.02	0.06	0.0702	0.03	−1.27	4.0	1.58	−1.20
Cambodia	1.6	0.02	0.10	8.4	0.13	0.63	462.6	7.09	0.62
Cameroon	2.5	0.02	0.02	9.6	0.09	2.37	545.2	5.17	2.44
Canada	66.3	0.49	0.90	10.7	0.08	0.42	575.7	4.44	0.46
Central African Republic	0.6	0.03	0.03	0.9	0.05	0.79	48.3	2.88	0.79
Chad	1.1	0.02	−0.03	2.9	0.07	2.89	166.3	3.71	2.96
Chile	20.9	0.30	0.10	7.1	0.10	2.54	382.6	5.40	2.54
China	5042.7	0.98	2.23	666.6	0.13	0.27	36069.8	7.08	0.29
Colombia	46.4	0.24	0.07	20.3	0.11	−1.56	1109.1	5.82	−1.56
Comoros	0.0885	0.03	0.10	0.2	0.06	0.61	9.2	3.43	0.59
Congo	0.7	0.03	−0.00347	1.3	0.07	0.89	72.0	3.70	0.91
Cook Islands	0.00148	0.03	−0.00604	0.00259	0.04	0.25	0.1	2.24	0.25
Costa Rica	5.3	0.28	−1.26	1.9	0.10	−0.32	103.9	5.47	−0.30
Côte d’Ivoire	1.6	0.02	−0.07	8.2	0.09	2.79	469.2	4.85	2.83
Croatia	3.4	0.22	0.04	0.5	0.03	−3.23	27.5	1.85	−3.24
Cuba	19.3	0.53	−0.33	7.1	0.19	0.07	377.9	10.35	0.03
Cyprus	1.5	0.24	−0.12	0.4	0.06	−1.37	19.3	3.27	−1.39
Czechia	34.4	0.83	0.71	0.9	0.02	−3.81	51.7	1.34	−3.68
Democratic People's Republic of Korea	6.2	0.06	0.24	15.6	0.15	0.83	839.7	8.12	0.82
Democratic Republic of the Congo	10.3	0.03	0.04	13.9	0.05	1.23	758.6	2.79	1.23
Denmark	17.9	0.94	−0.32	0.7	0.04	−1.68	36.8	1.96	−1.64
Djibouti	0.2	0.03	−0.08	0.3	0.06	1.34	15.9	3.13	1.35
Dominica	0.00616	0.03	−0.03	0.017	0.07	1.47	0.9	3.81	1.50
Dominican Republic	1.1	0.03	0.05	10.2	0.24	3.30	549.4	13.06	3.31
Ecuador	9.1	0.14	−0.85	11.6	0.17	3.44	638.5	9.46	3.48
Egypt	25.5	0.07	0.09	12.4	0.03	8.17	689.0	1.80	8.13
El Salvador	5.5	0.24	0.02	1.9	0.09	0.43	105.4	4.63	0.41
Equatorial Guinea	0.2	0.03	0.09	0.3	0.06	1.01	16.0	3.19	1.02
Eritrea	0.8	0.03	0.10	1.5	0.07	1.47	82.0	3.61	1.49
Estonia	1.6	0.33	−0.02	0.3	0.06	−3.44	15.1	3.21	−3.42
Eswatini	2.7	0.66	0.20	0.2	0.05	2.48	11.3	2.76	2.41
Ethiopia	17.1	0.05	0.03	15.9	0.05	−0.12	879.3	2.54	−0.10
Fiji	0.09	0.03	0.02	0.2	0.06	−0.44	11.0	3.17	−0.41
Finland	15.5	0.81	0.02	0.8	0.04	−1.02	41.8	2.29	−0.95
France	194.1	0.88	−0.14	13.5	0.06	−1.24	730.4	3.47	−1.23
Gabon	0.2	0.03	−0.04	0.4	0.06	0.76	20.7	3.40	0.75
Gambia	3.8	0.48	0.65	0.8	0.10	2.98	44.2	5.67	3.01
Georgia	0.4	0.04	0.03	4.7	0.37	12.29	248.4	19.51	12.23
Germany	167.9	0.57	0.94	10.6	0.04	−1.26	567.8	1.99	−1.25
Ghana	2.9	0.02	0.00391	9.0	0.07	0.52	515.2	4.17	0.54
Greece	31.0	0.81	0.02	3.0	0.08	−0.00615	157.6	4.50	−0.04
Greenland	0.5	2.33	−0.02	0.00251	0.01	−1.44	0.2	0.78	−1.25
Grenada	0.00981	0.03	−0.00754	0.0387	0.11	8.70	2.1	5.71	8.68
Guam	0.014	0.03	−0.00255	0.0442	0.08	3.61	2.3	4.31	3.67
Guatemala	13.4	0.24	0.02	7.6	0.14	−1.41	412.0	7.38	−1.41
Guinea	0.7	0.02	−0.25	3.1	0.08	2.70	178.9	4.30	2.76
Guinea-Bissau	0.2	0.02	−0.00207	0.7	0.11	2.51	41.6	5.98	2.59
Guyana	0.073	0.03	−0.00555	0.4	0.14	9.29	20.3	7.46	9.25
Haiti	1.3	0.03	0.01	5.1	0.10	1.37	273.8	5.64	1.39
Honduras	8.6	0.24	0.01	3.1	0.09	−0.88	165.8	4.65	−0.92
Hungary	13.3	0.35	−0.09	1.3	0.03	−4.91	66.4	1.88	−4.85
Iceland	1.0	0.82	0.03	0.0365	0.03	0.87	2.0	1.61	0.84
India	440.2	0.08	−0.12	278.3	0.05	0.46	14954.4	2.71	0.43
Indonesia	45.3	0.04	0.03	144.7	0.13	0.73	7870.3	6.94	0.72
Iran (Islamic Republic of)	85.9	0.22	−0.24	1.9	0.00486	0.17	109.3	0.28	0.18
Iraq	10.9	0.07	0.12	11.3	0.07	−0.10	623.1	4.09	−0.11
Ireland	26.0	1.33	0.00463	1.5	0.08	−1.00	78.9	4.34	−1.01
Israel	27.9	0.84	0.05	2.0	0.06	−0.69	111.1	3.41	−0.71
Italy	192.2	0.96	0.62	9.8	0.05	−1.43	529.7	2.82	−1.42
Jamaica	0.2	0.02	−0.52	0.4	0.04	0.15	20.6	1.90	0.09
Japan	33.4	0.09	0.71	10.7	0.03	0.01	560.7	1.41	0.00711
Jordan	13.3	0.28	−1.18	2.5	0.05	1.17	142.2	2.97	1.17
Kazakhstan	2.5	0.04	−0.02	10.3	0.14	0.42	543.7	7.50	0.37
Kenya	28.5	0.16	0.53	8.5	0.05	1.52	463.2	2.78	1.49
Kiribati	0.0121	0.03	0.00509	0.0122	0.03	0.36	0.6	1.48	0.36
Kuwait	1.2	0.04	−0.31	0.6	0.02	1.86	34.7	1.35	1.86
Kyrgyzstan	0.9	0.04	−1.03	6.7	0.27	5.39	360.0	14.68	5.38
Lao People's Democratic Republic	0.7	0.02	0.08	3.3	0.12	0.39	182.3	6.39	0.43
Latvia	1.7	0.27	0.29	0.7	0.11	−1.78	36.6	5.69	−1.81
Lebanon	7.3	0.31	0.13	0.7	0.03	0.23	38.1	1.63	0.26
Lesotho	4.4	0.66	0.24	0.3	0.05	3.86	17.9	2.73	3.75
Liberia	0.5	0.02	0.02	1.4	0.08	3.15	77.8	4.36	3.22
Libya	0.5	0.02	−0.00499	0.1	0.00351	5.65	6.1	0.19	5.53
Lithuania	3.2	0.36	0.04	0.7	0.08	−1.27	37.5	4.18	−1.29
Luxembourg	2.2	0.82	0.04	0.0813	0.03	−2.94	4.4	1.71	−2.92
Madagascar	3.0	0.03	−0.04	4.6	0.05	0.82	253.6	2.81	0.83
Malawi	1.2	0.02	−0.26	3.6	0.06	1.68	199.4	3.38	1.67
Malaysia	11.7	0.09	0.12	9.6	0.07	−0.91	523.2	3.98	−0.97
Maldives	0.0731	0.03	0.27	0.064	0.02	−0.10	3.5	1.19	−0.11
Mali	51.3	0.75	0.45	4.5	0.07	1.98	256.5	4.04	2.04
Malta	1.3	0.79	0.16	0.0907	0.06	0.70	4.8	2.94	0.62
Marshall Islands	0.00587	0.03	0.01	0.0337	0.15	1.12	1.7	8.02	1.13
Mauritania	0.3	0.02	−0.02	0.9	0.07	2.04	52.1	3.93	2.08
Mauritius	0.1	0.02	0.02	0.2	0.04	−1.02	11.2	2.30	−1.01
Mexico	156.8	0.32	0.01	52.0	0.10	−1.89	2824.2	5.72	−1.86
Micronesia (Federated States of)	0.01	0.03	−0.01	0.0555	0.16	0.90	2.9	8.16	0.93
Monaco	0.0925	0.81	−0.01	0.00523	0.05	0.26	0.3	2.64	0.23
Mongolia	0.5	0.04	0.02	1.2	0.09	8.44	62.4	4.80	8.41
Montenegro	0.8	0.33	0.03	0.0777	0.03	−1.03	4.2	1.83	−1.02
Morocco	2.8	0.02	−0.07	0.3	0.00191	3.75	15.1	0.11	3.59
Mozambique	2.9	0.03	−0.00217	5.9	0.07	2.19	325.1	3.78	2.19
Myanmar	5.1	0.02	0.03	22.8	0.11	−0.47	1258.4	6.03	−0.48
Namibia	4.6	0.53	0.53	0.3	0.04	1.62	19.1	2.21	1.57
Nauru	0.0011	0.03	−0.000054	0.00627	0.17	0.80	0.3	8.73	0.82
Nepal	1.9	0.02	0.02	4.9	0.04	0.61	264.8	2.38	0.61
Netherlands	88.5	1.59	0.32	1.9	0.03	−0.63	106.8	1.96	−0.59
New Zealand	146.7	8.13	−0.03	0.8	0.05	1.75	50.3	2.76	1.38
Nicaragua	6.2	0.25	0.08	2.0	0.08	0.03	108.2	4.25	0.01
Niger	1.6	0.02	0.01	3.3	0.06	2.01	188.8	3.14	2.08
Nigeria	19.9	0.03	0.01	46.8	0.07	2.78	2666.5	3.98	2.83
Niue	0.000141	0.03	−0.00118	0.000749	0.13	0.45	0.0388	6.79	0.49
North Macedonia	3.0	0.33	0.03	0.4	0.05	−2.47	23.3	2.59	−2.46
Northern Mariana Islands	0.00422	0.03	0.00207	0.0281	0.16	1.11	1.4	8.25	1.18
Norway	17.9	0.93	0.02	0.4	0.02	−0.55	22.7	1.21	−0.52
Oman	5.7	0.22	−0.19	0.6	0.02	0.72	35.1	1.39	0.76
Pakistan	23.2	0.03	−0.45	48.0	0.06	0.57	2636.2	3.18	0.59
Palau	0.00164	0.03	0.00693	0.0227	0.30	0.47	1.2	15.86	0.55
Palestine	0.8	0.05	−0.00276	1.1	0.06	0.36	65.3	3.60	0.37
Panama	3.3	0.21	−1.80	0.7	0.04	−0.26	37.7	2.45	−0.25
Papua New Guinea	1.0	0.03	0.02	3.7	0.10	0.91	196.2	5.44	0.94
Paraguay	0.4	0.02	−0.00118	3.1	0.11	0.73	165.0	6.02	0.73
Peru	4.2	0.03	−1.09	13.7	0.10	0.71	743.8	5.27	0.71
Philippines	70.9	0.16	0.50	49.9	0.12	−0.41	2755.1	6.52	−0.43
Poland	38.3	0.25	0.20	1.4	0.00884	−9.37	75.9	0.48	−9.26
Portugal	29.2	0.74	1.32	2.8	0.08	−1.90	150.3	4.16	−1.95
Puerto Rico	0.3	0.03	0.02	1.9	0.17	5.45	98.0	8.94	5.45
Qatar	0.3	0.02	−0.05	0.3	0.01	2.08	15.2	0.79	2.12
Republic of Korea	15.7	0.08	0.43	8.5	0.04	−3.46	451.6	2.33	−3.47
Republic of Moldova	4.8	0.31	0.02	1.4	0.09	−1.14	74.2	4.82	−1.18
Romania	10.4	0.14	−0.04	6.5	0.09	−2.40	336.8	4.92	−2.41
Russian Federation	174.4	0.31	0.43	58.4	0.10	−1.40	3060.5	5.26	−1.43
Rwanda	1.5	0.03	0.02	2.5	0.06	−0.44	139.4	3.06	−0.43
Saint Kitts and Nevis	0.00596	0.03	0.01	0.0354	0.15	5.73	1.9	7.91	5.73
Saint Lucia	0.0173	0.03	0.00572	0.0474	0.07	4.02	2.5	3.57	4.02
Saint Vincent and the Grenadines	0.0105	0.03	0.02	0.0701	0.17	6.44	3.7	8.90	6.38
Samoa	0.0187	0.03	−0.05	0.0867	0.14	1.13	4.5	7.10	1.14
San Marino	0.0871	0.80	−0.01	0.00288	0.03	−0.32	0.2	1.58	−0.31
Sao Tome and Principe	0.0185	0.02	0.03	0.00884	0.01	0.22	0.5	0.65	0.23
Saudi Arabia	17.2	0.08	−0.33	6.9	0.03	3.31	371.0	1.76	3.27
Senegal	6.6	0.12	0.06	4.3	0.08	2.71	243.6	4.79	2.77
Serbia	10.1	0.29	−0.05	2.3	0.07	−2.02	120.8	3.56	−2.03
Seychelles	0.00594	0.02	0.00775	0.0344	0.08	0.28	1.8	4.31	0.30
Sierra Leone	0.7	0.02	0.01	2.1	0.07	3.23	122.5	4.09	3.29
Singapore	4.0	0.14	0.33	0.4	0.01	−4.03	19.9	0.78	−3.96
Slovakia	10.7	0.46	−0.43	1.0	0.04	−2.19	51.7	2.38	−2.14
Slovenia	4.5	0.56	1.05	0.1	0.01	−5.15	6.0	0.79	−5.04
Solomon Islands	0.0647	0.03	−0.00955	0.3	0.15	1.64	17.9	7.80	1.65
Somalia	2.1	0.03	0.07	2.9	0.05	0.81	155.9	2.50	0.83
South Africa	584.5	2.51	0.38	18.9	0.08	1.09	1025.7	4.39	0.98
South Sudan	0.9	0.03	−0.18	1.3	0.05	1.23	69.0	2.52	1.24
Spain	205.8	1.16	0.93	9.7	0.06	−2.70	518.7	3.26	−2.67
Sri Lanka	1.8	0.02	−0.34	8.6	0.11	−3.11	468.1	5.77	−3.14
Sudan	6.6	0.04	−0.07	0.3	0.002	4.26	17.3	0.11	4.03
Suriname	0.0522	0.03	−0.00301	0.1	0.07	1.88	7.9	3.80	1.85
Sweden	32.8	0.93	0.03	2.0	0.06	−0.38	108.0	3.12	−0.31
Switzerland	40.5	1.20	−0.33	1.0	0.03	−2.14	55.6	1.73	−2.15
Syrian Arab Republic	2.0	0.04	−0.22	0.0142	0.000307	−0.44	0.9	0.02	−0.42
Taiwan (Province of China)	5.5	0.06	0.46	7.5	0.08	−0.52	390.1	4.06	−0.59
Tajikistan	1.4	0.04	0.00663	2.3	0.06	−0.82	127.3	3.51	−0.80
Thailand	76.6	0.33	1.41	44.9	0.18	0.31	2405.6	9.92	0.32
Timor-Leste	0.1	0.02	−0.02	0.4	0.10	1.12	23.5	5.47	1.13
Togo	0.7	0.02	−0.02	2.3	0.08	2.36	129.5	4.58	2.43
Tokelau	0.000121	0.03	0.00331	0.000638	0.14	0.56	0.0336	7.20	0.62
Tonga	0.0091	0.03	0.04	0.1	0.43	−0.02	6.9	21.69	0.02
Trinidad and Tobago	1.6	0.30	1.24	0.3	0.06	0.86	18.2	3.30	0.89
Tunisia	4.0	0.08	−0.30	0.0884	0.00186	3.52	5.1	0.11	3.10
Turkey	126.3	0.38	−0.03	28.2	0.08	−1.15	1549.6	4.70	−1.14
Turkmenistan	0.7	0.04	0.02	4.3	0.22	10.56	231.6	12.05	10.52
Tuvalu	0.00119	0.03	0.00869	0.00657	0.16	0.88	0.3	8.25	0.93
Uganda	40.6	0.32	0.56	5.9	0.05	1.73	326.7	2.74	1.73
Ukraine	67.6	0.39	0.03	27.9	0.16	−0.34	1466.5	8.56	−0.35
United Arab Emirates	4.1	0.06	0.04	2.5	0.05	−0.12	132.2	2.93	0.10
United Kingdom	236.2	1.00	0.30	17.6	0.07	0.95	954.6	4.10	0.91
United Republic of Tanzania	5.9	0.03	0.02	9.5	0.05	0.75	524.1	2.93	0.76
United States Virgin Islands	0.00613	0.03	0.02	0.023	0.10	4.04	1.3	5.42	4.27
United States of America	22532.5	19.16	1.73	87.9	0.08	−1.63	5851.8	5.10	−1.12
Uruguay	5.9	0.47	0.09	0.9	0.07	0.79	46.8	3.82	0.81
Uzbekistan	4.9	0.04	0.01	13.7	0.10	2.31	743.9	5.54	2.31
Vanuatu	0.0297	0.03	0.000247	0.1	0.13	1.04	7.0	6.78	1.06
Venezuela (Bolivarian Republic of)	25.2	0.24	0.03	22.7	0.22	0.92	1220.2	12.31	0.99
Viet Nam	6.7	0.02	−0.33	47.5	0.12	0.43	2549.8	6.31	0.40
Yemen	5.2	0.05	0.06	0.2	0.00142	4.58	9.0	0.08	4.21
Zambia	2.1	0.03	0.03	4.2	0.07	1.15	230.0	3.76	1.14
Zimbabwe	13.0	0.26	−0.12	2.7	0.05	3.16	145.8	2.91	3.14

Abbreviations: ASDR, age-standardized death rate; ASIR, age-standardized incidence rate; DALY, disability-adjusted life year; EAPC, estimated annual percentage change.

### Mortality trends in the United States, 1999–2023

**Figure 4:**
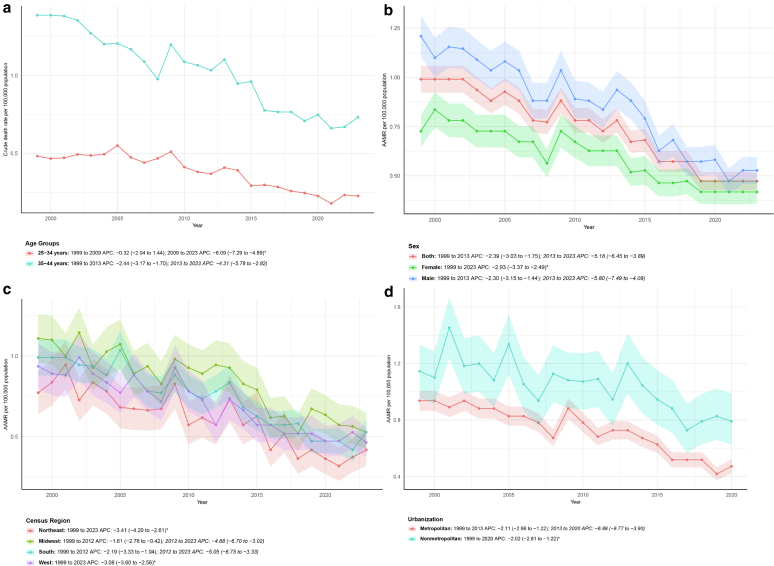
**Trends in mortality rates of malignant melanoma of skin among younger adults in the USA, 1999–2023.** (**a**) Age-specific crude mortality rates for ages 25–34 and 35–44 years. (**b–d**) Age-adjusted mortality rates for sex, census region, and urbanization strata within the 25–44-year population. Shaded areas represent 95% confidence intervals. USA, United States of America.

In the United States, CDC WONDER data showed a marked decline in melanoma mortality among adults aged 25–44 years from 1999 to 2023. The rate decreased from 0.99 (95% CI = 0.92–1.06) per 100,000 population to 0.47 (95% CI = 0.43–0.52), with an average annual percent change of −3.56% (95% CI = −4.18% to −2.94%) (Table 8). Age-adjusted mortality rates declined for the overall 25–44-year population and for subgroups defined by sex, region, and urbanization level; crude age-specific mortality rates also declined for the 25–34-year and 35–44-year strata. The decline was more pronounced in men (average annual percent change = −3.77% vs −2.93%), although male mortality remained higher throughout the study period. The decline accelerated in recent years in the South and in metropolitan areas (Figure 4). By contrast, mortality rates for other malignant neoplasms of the skin and for peripheral and cutaneous T-cell lymphomas were low (0.05 per 100,000 in both 1999 and 2023) and showed no significant temporal trend (Tables 9 and 10).

**Table 8:** Trends in Mortality Burden of Malignant Melanoma of Skin among Younger Adults Aged 25–44 Years in the United States, 1999–2023

Measure	Deaths			Mortality Rate		
	1999	2023	Percent Change (%)	1999 (95% CI)	2023 (95% CI)	AAPC (95% CI)
Sex						
Both	819	428	−47.74	0.99 (0.92–1.06)	0.47 (0.43–0.52)	−3.56 (−4.18 to −2.94)
Female	312	193	−38.14	0.73 (0.65–0.81)	0.42 (0.36–0.48)	−2.93 (−3.37 to −2.49)
Male	507	235	−53.65	1.21 (1.10–1.31)	0.53 (0.46–0.59)	−3.77 (−4.59 to −2.95)
Age						
25–34 y	194	103	−46.91	0.48 (0.41–0.55)	0.23 (0.18–0.27)	−3.73 (−4.67 to −2.78)
35–44 y	625	325	−48.00	1.39 (1.28–1.50)	0.73 (0.65–0.81)	−3.22 (−3.93 to −2.51)
Census region						
Northeast	128	61	−52.34	0.77 (0.64–0.91)	0.42 (0.32–0.54)	−3.41 (−4.20 to −2.61)
Midwest	212	88	−58.49	1.11 (0.96–1.26)	0.53 (0.42–0.65)	−3.12 (−4.12 to −2.11)
South	303	176	−41.91	0.99 (0.88–1.10)	0.53 (0.45–0.61)	−3.51 (−4.46 to −2.56)
West	176	103	−41.48	0.94 (0.80–1.07)	0.46 (0.37–0.55)	−3.08 (−3.60 to −2.56)
Urbanization						
Metropolitan	677	342	−32.88	0.94 (0.87–1.01)	0.47 (0.42–0.52)	−3.73 (−4.80 to −2.64)
Nonmetropolitan	142	86	−24.56	1.15 (0.96–1.33)	0.79 (0.63–0.99)	−2.02 (−2.81 to −1.22)
Race						
Hispanic	19	43	126.32	NA (0.13–0.33)	0.26 (0.19–0.36)	NA
NH Black	NA	NA	NA	NA	NA	NA
NH White	787	370	−52.99	1.35 (1.26–1.45)	0.78 (0.70–0.86)	−2.75 (−3.51 to −1.98)
NH other	NA	NA	NA	NA	NA	NA

Analyses of younger adults were restricted to the 2 standard CDC 10-year age groups (25–34 and 35–44 years), which together constitute the 25–44-year population. Mortality rates for the overall 25–44-year group and for subgroups defined by sex, census region, race and ethnicity, and urbanization level were age-adjusted mortality rates obtained directly from CDC WONDER; these rates are standardized by the NCHS to the projected year 2000 United States standard population using the direct method. Mortality rates for the 25–34-year and 35–44-year age groups are crude age-specific mortality rates because CDC WONDER does not calculate age-adjusted rates when data are stratified by age. Race and ethnicity categories were analyzed to describe subgroup-specific mortality patterns and potential disparities using categories available in CDC WONDER; this analysis was not funder-mandated. Race and ethnicity classifications were based on death certificate demographic information as compiled by NCHS/CDC WONDER and were not assigned by the investigators. Categories were analyzed as provided by CDC WONDER and grouped as Hispanic, non-Hispanic Black, non-Hispanic White, and non-Hispanic Other. Cell counts suppressed by CDC WONDER are reported as NA.

Abbreviations: AAPC, average annual percentage change; CDC, Centers for Disease Control and Prevention; CDC WONDER, Centers for Disease Control and Prevention Wide-ranging Online Data for Epidemiologic Research; CI, confidence interval; NA, not available; NCHS, National Center for Health Statistics; NH, non-Hispanic.

**Table 9:** Trends in Mortality Burden of Other Malignant Neoplasms of Skin among Younger Adults Aged 25–44 Years in the United States, 1999–2023

Measure	Deaths			Mortality Rate		
	1999	2023	Percent Change (%)	1999 (95% CI)	2023 (95% CI)	AAPC (95% CI)
Sex						
Both	68	60	−11.76	0.05 (0.04–0.07)	0.05 (0.04–0.07)	0.00 (0.00–0.00)
Female	11	19	72.73	NA (0.00–0.00)	NA (0.03–0.09)	NA
Male	57	41	−28.07	0.11 (0.08–0.14)	0.11 (0.08–0.15)	−1.89 (−3.64 to −0.10)
Age						
25–34 y	NA	NA	NA	NA	NA	NA
35–44 y	62	51	−17.74	0.14 (0.11–0.18)	0.11 (0.09–0.15)	−0.85 (−1.87 to 0.19)
Census region						
Northeast	10	NA	NA	NA (0.02–0.11)	NA	NA
Midwest	11	11	0.00	NA (0.03–0.10)	NA (0.03–0.10)	NA
South	35	30	−14.29	0.11 (0.08–0.15)	0.05 (0.04–0.08)	NA
West	12	12	0.00	NA (0.03–0.10)	NA (0.03–0.10)	NA
Urbanization						
Metropolitan	57	46	−19.30	0.05 (0.04–0.07)	0.05 (0.04–0.07)	NA
Nonmetropolitan	11	14	27.27	NA (0.05–0.19)	NA	NA
Race						
Hispanic	NA	NA	NA	NA	NA	NA
NH Black	11	10	−9.09	NA (0.05–0.21)	NA (0.02–0.11)	NA
NH White	53	40	−24.53	0.11 (0.08–0.14)	0.05 (0.04–0.08)	−2.45 (−2.45 to −2.45)
NH other	NA	NA	NA	NA	NA	NA

Analyses of younger adults were restricted to the 2 standard CDC 10-year age groups (25–34 and 35–44 years), which together constitute the 25–44-year population. Rates for the overall 25–44-year group and subgroups defined by sex, region, race, and urbanization level were age-adjusted mortality rates obtained directly from CDC WONDER; these rates are standardized by NCHS to the projected year 2000 United States standard population using the direct method. Rates for the 25–34-year and 35–44-year age groups are crude age-specific mortality rates because CDC WONDER does not calculate age-adjusted rates when data are stratified by age. "Mortality rate" denotes age-adjusted mortality rates for the overall 25–44-year group and subgroups defined by sex, region, race, and urbanization level and crude age-specific mortality rates for the 25–34-year and 35–44-year strata. Race and ethnicity categories were analyzed to describe subgroup-specific mortality patterns and potential disparities using categories available in CDC WONDER; this analysis was not funder-mandated. Race and ethnicity classifications were based on death certificate demographic information as compiled by NCHS/CDC WONDER and were not assigned by the investigators. Categories were analyzed as provided by CDC WONDER and grouped as Hispanic, non-Hispanic Black, non-Hispanic White, and non-Hispanic Other. Cell counts suppressed by CDC WONDER are reported as NA.

Abbreviations: AAPC, average annual percentage change; CDC, Centers for Disease Control and Prevention; CDC WONDER, Centers for Disease Control and Prevention Wide-ranging Online Data for Epidemiologic Research; CI, confidence interval; NA, not available; NCHS, National Center for Health Statistics; NH, non-Hispanic.

**Table 10:** Trends in Mortality Burden of Peripheral and Cutaneous T-Cell Lymphomas among Younger Adults Aged 25–44 Years in the United States, 1999–2023

Measure	Deaths			Mortality Rate		
	1999	2023	Percent Change (%)	1999 (95% CI)	2023 (95% CI)	AAPC (95% CI)
Sex						
Both	53	71	33.96	0.05 (0.04–0.07)	0.05 (0.04–0.07)	−0.64 (−2.93 to 1.71)
Female	19	29	52.63	NA (0.03–0.09)	0.05 (0.03–0.09)	NA
Male	34	42	23.53	0.10 (0.07–0.14)	0.05 (0.04–0.08)	−1.16 (−4.68 to 2.48)
Age						
25–34 y	14	22	57.14	NA	0.05 (0.03–0.07)	NA
35–44 y	39	49	25.64	0.09 (0.06–0.12)	0.11 (0.08–0.15)	0.60 (−0.97 to 2.20)
Census region						
Northeast	15	11	−26.67	NA (0.05–0.17)	NA (0.05–0.18)	NA
Midwest	12	15	25.00	NA (0.03–0.10)	NA (0.05–0.17)	NA
South	13	28	115.38	NA (0.02–0.10)	0.05 (0.03–0.08)	NA
West	13	17	30.77	NA (0.05–0.17)	NA (0.06–0.16)	NA
Urbanization						
Metropolitan	43	67	55.81	0.05 (0.04–0.08)	0.10 (0.08–0.13)	0.41 (−1.70 to 2.56)
Nonmetropolitan	10	NA	NA	NA (0.05–0.19)	NA	NA
Race						
Hispanic	NA	16	NA	NA	NA (0.06–0.16)	NA
NH Black	13	26	100.00	NA (0.05–0.20)	0.21 (0.14–0.31)	NA
NH White	29	24	−17.24	0.05 (0.03–0.08)	0.05 (0.03–0.09)	NA
NH other	NA	NA	NA	NA	NA	NA

Analyses of younger adults were restricted to the 2 standard CDC 10-year age groups (25–34 and 35–44 years), which together constitute the 25–44-year population. Rates for the overall 25–44-year group and subgroups defined by sex, region, race, and urbanization level were age-adjusted mortality rates obtained directly from CDC WONDER; these rates are standardized by NCHS to the projected year 2000 United States standard population using the direct method. Rates for the 25–34-year and 35–44-year age groups are crude age-specific mortality rates because CDC WONDER does not calculate age-adjusted rates when data are stratified by age. "Mortality rate" denotes age-adjusted mortality rates for the overall 25–44-year group and subgroups defined by sex, region, race, and urbanization level and crude age-specific mortality rates for the 25–34-year and 35–44-year strata. Race and ethnicity categories were analyzed to describe subgroup-specific mortality patterns and potential disparities using categories available in CDC WONDER; this analysis was not funder-mandated. Race and ethnicity classifications were based on death certificate demographic information as compiled by NCHS/CDC WONDER and were not assigned by the investigators. Categories were analyzed as provided by CDC WONDER and grouped as Hispanic, non-Hispanic Black, non-Hispanic White, and non-Hispanic Other. Cell counts suppressed by CDC WONDER are reported as NA.

Abbreviations: AAPC, average annual percentage change; CDC, Centers for Disease Control and Prevention; CDC WONDER, Centers for Disease Control and Prevention Wide-ranging Online Data for Epidemiologic Research; CI, confidence interval; NA, not available; NCHS, National Center for Health Statistics; NH, non-Hispanic.

### Age and regional patterns of skin cancer burden in younger adults

**Figure 5:**
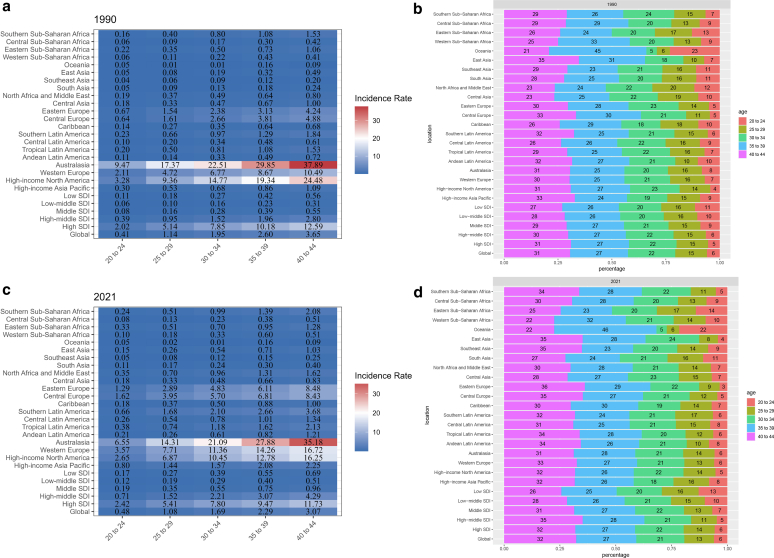
**Incidence rates and age distribution of malignant melanoma among younger adults across GBD regions and SDI quintiles, 1990 and 2021.** (**a, c**) Heatmaps show age-specific incidence rates (per 100,000 population) in (**a**) 1990 and (**c**) 2021. (**b, d**) Stacked bar charts show the proportional distribution of cases across age groups (20–24, 25–29, 30–34, 35–39, 40–44 years) in (**b**) 1990 and (**d**) 2021. GBD, Global Burden of Disease; SDI, Sociodemographic Index.

**Figure 6:**
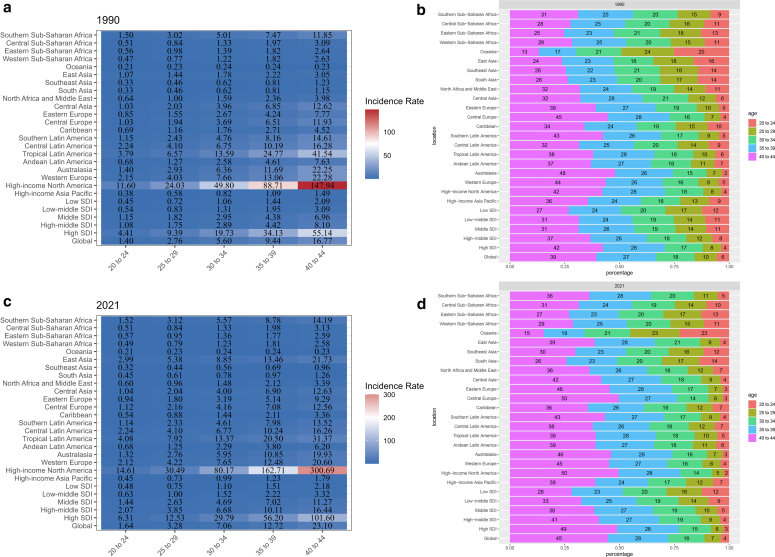
**Incidence rates and age distribution of basal cell carcinoma among younger adults across GBD regions and SDI quintiles, 1990 and 2021.** (**a, c**) Heatmaps show age-specific incidence rates (per 100,000 population) in (**a**) 1990 and (**c**) 2021. (**b, d**) Stacked bar charts show the proportional distribution of cases across age groups (20–24, 25–29, 30–34, 35–39, 40–44 years) in (**b**) 1990 and (**d**) 2021. GBD, Global Burden of Disease; SDI, Sociodemographic Index.

**Figure 7:**
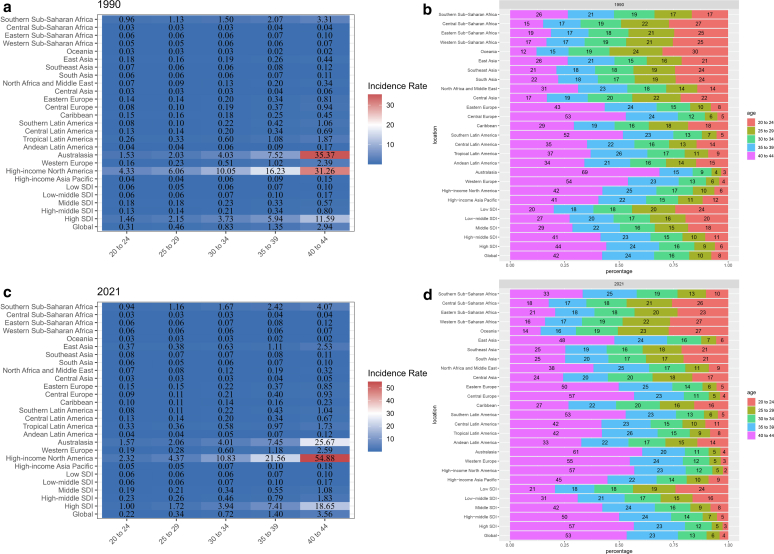
**Incidence rates and age distribution of squamous cell carcinoma among younger adults across GBD regions and SDI quintiles, 1990 and 2021.** (**a, c**) Heatmaps show age-specific incidence rates (per 100,000 population) in (**a**) 1990 and (**c**) 2021. (**b, d**) Stacked bar charts show the proportional distribution of cases across age groups (20–24, 25–29, 30–34, 35–39, 40–44 years) in (**b**) 1990 and (**d**) 2021. GBD, Global Burden of Disease; SDI, Sociodemographic Index.

**Figure 8:**
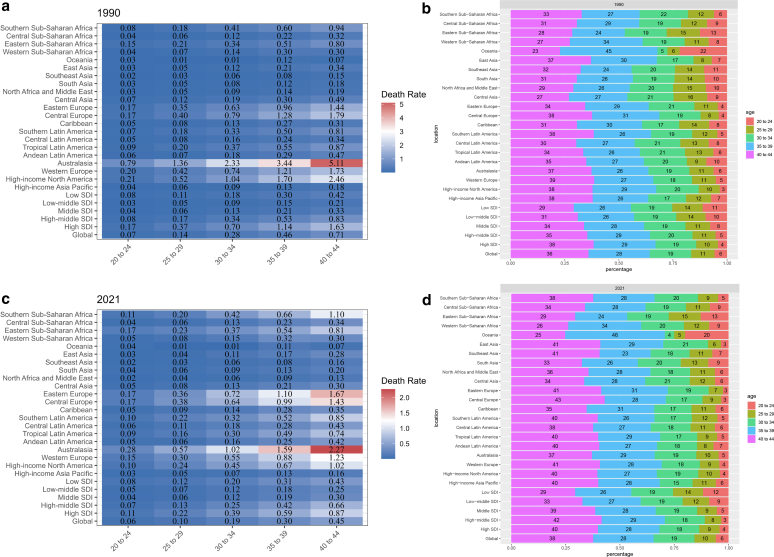
**Death rates and age distribution of malignant melanoma among younger adults across GBD regions and SDI quintiles, 1990 and 2021.** (**a, c**) Heatmaps show age-specific death rates (per 100,000 population) in (**a**) 1990 and (**c**) 2021. (**b, d**) Stacked bar charts show the proportional distribution of deaths across age groups (20–24, 25–29, 30–34, 35–39, 40–44 years) in (**b**) 1990 and (**d**) 2021. GBD, Global Burden of Disease; SDI, Sociodemographic Index.

**Figure 9:**
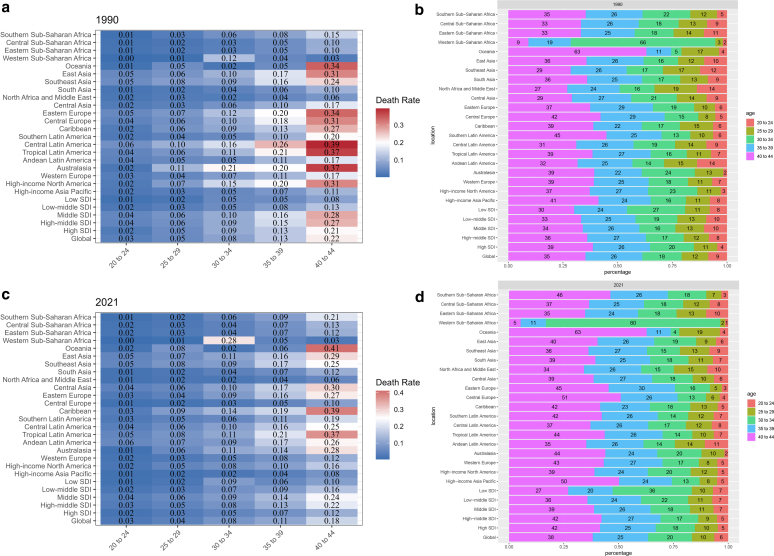
**Death rates and age distribution of squamous cell carcinoma among younger adults across GBD regions and SDI quintiles, 1990 and 2021.** (**a, c**) Heatmaps show age-specific death rates (per 100,000 population) in (**a**) 1990 and (**c**) 2021. (**b, d**) Stacked bar charts show the proportional distribution of deaths across age groups (20–24, 25–29, 30–34, 35–39, 40–44 years) in (**b**) 1990 and (**d**) 2021. GBD, Global Burden of Disease; SDI, Sociodemographic Index.

Between 1990 and 2021, the incidence of all investigated skin cancers among younger adults showed an age-related increase, with the highest rates observed in the 40–44 years age group (3.07, 23.10, and 3.56 per 100,000 for melanoma, BCC, and SCC in 2021, respectively). In 2021, the geographic and socioeconomic patterns varied by cancer type. For melanoma, the highest ASIRs and age-standardized mortality rates were observed in Australasia and in high-SDI regions. For BCC and SCC, the incidence was highest in high-income North America and other high-SDI regions. In contrast, SCC mortality rates were greatest in Oceania and in middle-SDI regions, differing from the incidence distribution (Figure 5, Figure 6, Figure 7, Figure 8, Figure 9).

### Joinpoint analysis of incidence trends in skin cancer among younger adults

**Figure 10:**
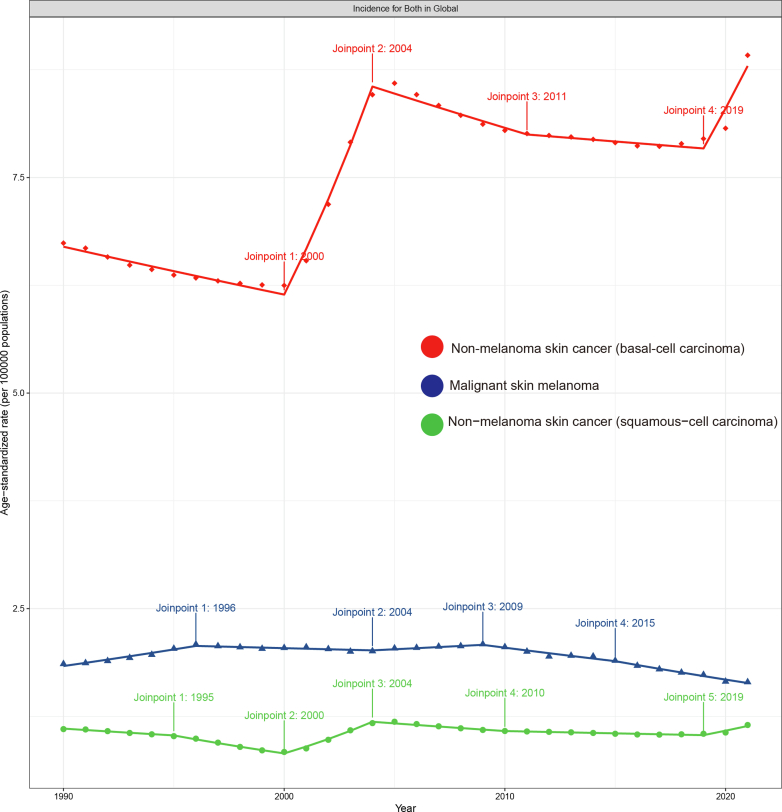
**Joinpoint regression analysis of skin cancer ASIR from 1990 to 2021.** ASIR, age-standardized incidence rate.

From 1990 to 2021, ASIR trends differed by cancer type. For melanoma, the ASIR showed a modest overall decline. Joinpoint analysis identified 3 periods: an increase from 1990 to 1996, stability from 1996 to 2009, and a decline that accelerated from 2015 to 2021. In contrast, BCC showed a steady upward trend throughout the study period. For SCC, the overall trend was not significant, but fluctuations were observed, with increases from 2000 to 2004 and from 2019 to 2021 (Figure 10 and Table 11).

**Table 11:** Joinpoint Regression Analysis Results of Skin Cancer ASIR from 1990 to 2021

Cause	Start	End	APC (95% CI)	AAPC (95% CI)
Malignant skin melanoma	1990	1996	2.02 (1.58–2.48)	−0.37 (−0.43 to −0.31)
	1996	2004	−0.32 (−0.98 to 1.04)	
	2004	2009	0.63 (−0.45 to 1.43)	
	2009	2015	−1.58 (−1.89 to 0.56)	
	2015	2021	−2.37 (−3.19 to −2.01)	
Nonmelanoma skin cancer (basal cell carcinoma)	1990	2000	−0.86 (−1.17 to −0.64)	0.88 (0.79–0.95)
	2000	2004	8.64 (−0.56 to 9.15)	
	2004	2011	−0.95 (−1.67 to 8.16)	
	2011	2019	−0.26 (−0.62 to 0.44)	
	2019	2021	5.92 (3.99–7.14)	
Nonmelanoma skin cancer (squamous cell carcinoma)	1990	1995	−1.47 (−2.03 to −0.81)	0.09 (0–0.16)
	1995	2000	−4.43 (−5 to −3.97)	
	2000	2004	9.69 (9.15–10.26)	
	2004	2010	−1.57 (−2.31 to −1.19)	
	2010	2019	−0.5 (−0.77 to −0.07)	
	2019	2021	5.03 (3.17–6.16)	

Abbreviations: AAPC, average annual percent change; APC, annual percent change; ASIR, age-standardized incidence rate; CI, confidence interval.

### Frontier analysis of age-standardized DALY rates in younger adults

**Figure 11:**
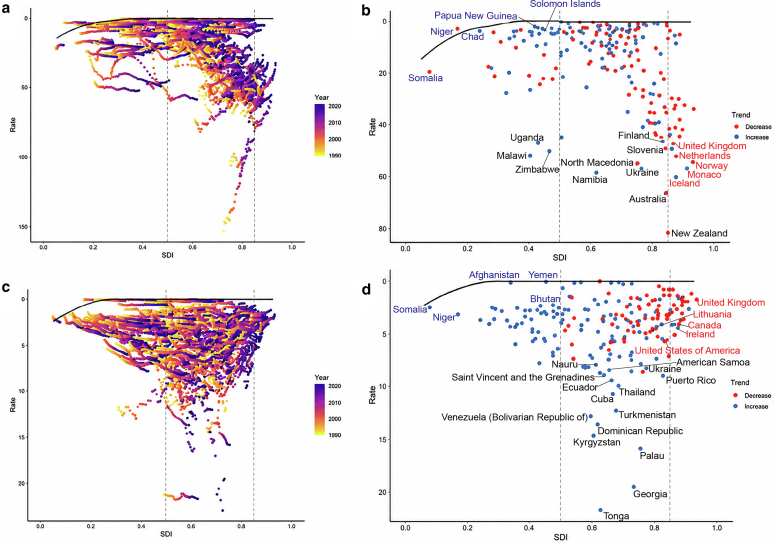
**Frontier analysis of skin cancer based on SDI and age-standardized DALY rate per 100,000 population.** (**a, c**) Temporal trends in age-standardized DALY rate relative to SDI of (**a**) malignant melanoma and (**c**) squamous cell carcinoma from 1990 to 2021. (**b, d**) Scatterplot of individual countries showing the age-standardized DALY rate relative to SDI of (**b**) malignant melanoma and (**d**) squamous cell carcinoma in 2021. The black frontier line represents the potentially achievable age-standardized DALY rate on the basis of SDI. Red dots show a decrease in age-standardized DALY rates from 1990 to 2021, whereas the blue dots indicate an increase over the same period. The top 15 countries with the largest effective difference are labeled in black. Countries and territories with low SDI (<0.5) of the top 5 with the lowest effective difference are labeled in blue. Countries and territories with high SDI (>0.85) of the top 5 with the highest effective difference are labeled in red. DALY, disability-adjusted life year; SDI, Sociodemographic Index.

**Figure 12:**
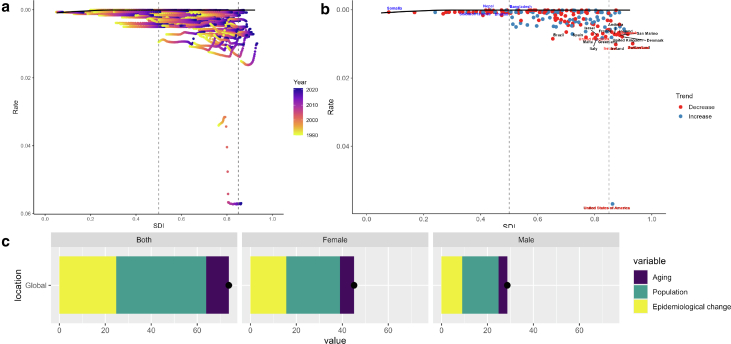
**Frontier analysis and decomposition analysis of basal cell carcinoma.** (**a**) Temporal trends in age-standardized DALY rate relative to SDI 1990 to 2021. (**b**) Scatterplot of individual countries showing the age-standardized DALY rate relative to SDI in 2021. (**c**) Decomposition of basal cell carcinoma changes. DALY, disability-adjusted life year; SDI, Sociodemographic Index.

Frontier analysis benchmarked country-level DALY rates against the lowest achievable rate (efficiency frontier) for each SDI level. For melanoma, most high-SDI countries were positioned near the efficiency frontier, and their DALY rates generally decreased from 1990 to 2021. Notable exceptions included Australia, New Zealand, and the Netherlands, which had substantially higher burdens. By contrast, many middle- and low-SDI countries experienced rising DALY rates. For SCC, many countries across all SDI levels were distant from the frontier, with most showing increasing DALY rates. For example, high-middle-SDI countries, such as Tonga and Georgia, had burdens far above the benchmark for their SDI level. For BCC, the burden was minimal, with the age-standardized DALY rates for nearly all countries situated on or very near the efficiency frontier (Figures 11 and 12a and b).

### Decomposition analysis of global skin cancer DALYs in younger adults

**Figure 13:**
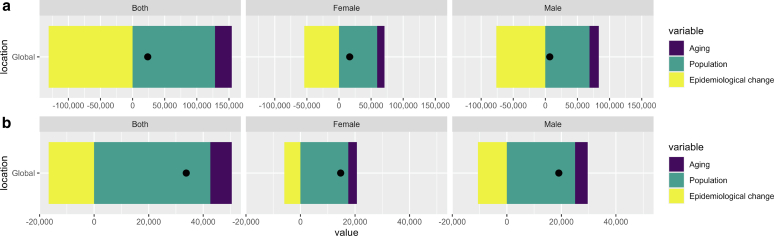
**Decomposition analysis of skin cancer burden.** Changes in DALYs (both sexes, women, and men) are decomposed into contributions from population growth (more people aged 20–44 years), population aging within this age range (a shift toward older ages), and epidemiological change (changes in age-specific DALY rates with population size and age structure held constant, reflecting changes in incidence and outcomes such as case fatality, stage at diagnosis, and severity due to prevention, earlier detection, or improved treatment). The black dot indicates the observed net change (the sum of all components). Percentage contributions are scaled to this net change; values >100% or ≤100% indicate that a single component would have produced a larger increase or decrease than observed but was partly offset by the other components. Positive values indicate increased DALYs (unfavorable), and negative values indicate reduced DALYs (favorable). (**a**) Malignant melanoma. (**b**) Squamous cell carcinoma. DALY, disability-adjusted life year.

Melanoma DALYs increased by 23,489, driven by population growth (546.5%) and aging (110.2%), but were offset by epidemiological improvements (−556.7%). SCC DALYs rose by 33,773, primarily owing to population growth, with partial mitigation from epidemiological factors. In contrast, the minimal increase in BCC DALYs reflected contributions from population growth (53.1%), aging (13.3%), and adverse epidemiological change (33.6%). These patterns were consistent across sexes (Figures 12c and 13).

### Health inequality in the burden of skin cancer among younger adults

**Figure 14:**
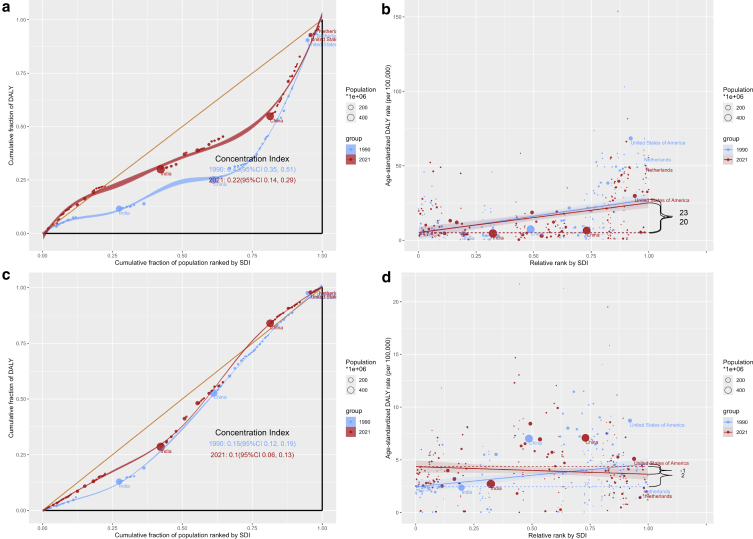
**Concentration curves (denoted in a and c) and health inequality regression curves (denoted in b and d) for skin cancer DALYs globally in 1990 and 2021.** (**a, b**) Malignant melanoma. (**c, d**) Squamous cell carcinoma. Larger circles indicate countries with higher populations. The red and blue lines represent linear trends for 1990 and 2021, respectively. DALY, disability-adjusted life year.

The burden of melanoma and SCC was concentrated in high-SDI countries, but disparities declined between 1990 and 2021. For melanoma, the concentration index declined from 0.43 (95% CI = 0.35–0.51) in 1990 to 0.22 (95% CI = 0.14–0.29) in 2021. The slope index of inequality also declined, from 23 to 20, although it continued to show a strong positive association between age-standardized DALY rates and SDI. For SCC, the concentration index fell from 0.15 (95% CI = 0.12–0.19) to 0.10 (95% CI = 0.06–0.13). The slope index of inequality shifted from positive to negative (2 in 1990 to −1 in 2021), suggesting that the absolute burden, although small, may have shifted toward lower-SDI regions (Figure 14).

### Global projections of skin cancer incidence in younger adults

**Figure 15:**
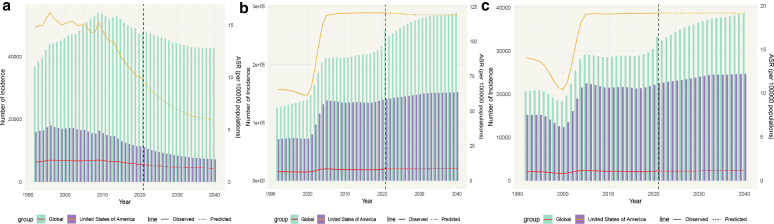
**Predicted trends in incidence numbers and age-standardized rates (per 100,000 population) of skin cancer from 2022 to 2040, based on Nordpred model.** The bar chart illustrates changes in numbers, whereas the curve depicts changes in age-standardized rates. (**a**) Malignant melanoma. (**b**) Basal cell carcinoma. (**c**) Squamous cell carcinoma. ASR, age-standardized rate.

**Figure 16:**
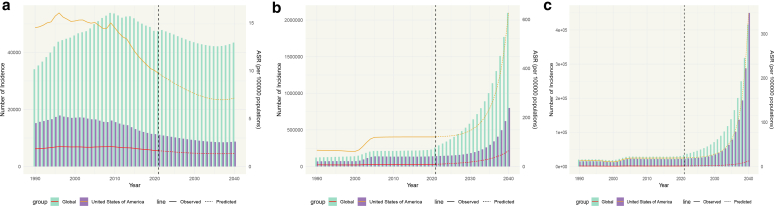
**Predicted trends in incidence numbers and age-standardized rates (per 100,000 population) of skin cancer from 2022 to 2040, based on BAPC model.** The bar chart illustrates changes in numbers, whereas the curve depicts changes in age-standardized rates. (**a**) Malignant melanoma. (**b**) Basal cell carcinoma. (**c**) Squamous cell carcinoma. ASR, age-standardized rate; BAPC, Bayesian age-period-cohort.

Future skin cancer incidence to 2040 for adults aged 20–44 years was estimated using the Nordpred (Figure 15) and Bayesian age-period-cohort (Figure 16) models. For melanoma, both models suggested a slow global decline in incident cases and ASIR, with a similar pattern in the United States. For BCC and SCC, both models indicated increasing global incidence. The Nordpred model suggested stabilization of ASIRs, whereas the Bayesian age-period-cohort model pointed to rising case numbers, although with varying magnitudes. In the United States, increases in nonmelanoma skin cancer (NMSC) appeared smaller than the corresponding global trends, with ASIRs remaining essentially stable.

## Discussion

Although previous studies indicate a rising global burden of skin cancer, driven largely by NMSC in older populations, our study—to our knowledge, the first comprehensive assessment in adults aged 20–44 years—reveals a more complex picture (Wang et al, 2025, Zhou et al, 2025). Although absolute numbers of incident cases, deaths, and DALYs in this younger group increased over the past 3 decades, their ASRs showed divergent trends: the ASIR for NMSC rose even as its DALY rate fell, whereas the ASR for malignant melanoma steadily decreased. This divergence stems from multifactorial drivers, primarily distinct etiologies. SCC is linked to cumulative UV exposure, whereas melanoma and BCC are more associated with intermittent exposure (Armstrong and Cust, 2017, Schadendorf et al, 2018). The sharp NMSC rise (2000–2005) paralleled dermoscopy adoption, likely enhancing the detection of subtle UV-related lesions. The decline in melanoma burden since 2010 may reflect the delayed impact of sun-safety campaigns and regulations on UV-emitting tanning devices (Del Mar, 1995, El Ghissassi et al, 2009, Reiter et al, 2019, Schmidt, 2012, Watts et al, 2018, Williams et al, 2018, Yélamos et al, 2019). This mirrors trends in Australia, where declining incidence in younger cohorts has been attributed to reduced UV exposure from behavioral changes (Whiteman et al, 2024).

The declining mortality coincided with advances in systemic treatment. Immune checkpoint inhibitors (eg, anti–CTLA-4 and anti–PD-1 agents) and targeted therapies (eg, BRAF and MEK inhibitors) have improved survival in advanced melanoma, with systemic therapies also benefiting advanced SCC (Curti and Faries, 2021, Wysong, 2023). Consistent with this, the global age-standardized death rate for SCC declined between 1990 and 2021 despite rising case numbers, suggesting that improvements in early detection and treatment have reduced case fatality in this population. Our analysis of CDC WONDER data reveals an accelerated decline in melanoma age-adjusted mortality rates among United States younger adults after 2012, coinciding with the introduction of these therapies and standardized staging and follow-up (Carlino et al, 2021). Similarly, a nationwide study from the Netherlands reported marked improvements in 5-year relative survival for advanced melanoma after the widespread adoption of systemic therapies after 2013 (Leeneman et al, 2021). In parallel, greater public awareness and enhanced screening have led to the diagnosis of melanoma at earlier stages, resulting in better prognoses and improved patient survival (Waseh and Lee, 2023). By contrast, mortality from rare malignancies such as peripheral and cutaneous T-cell lymphomas remained low and stable, but absolute deaths increased from 53 to 71. This discrepancy highlights the need for better classification and registration of rare tumors to assess their burden and the real-world effectiveness of new therapies in this population.

Age and sex differences were evident in the global distribution of skin cancer among younger adults. Disease burden increased with age, consistent with cumulative UV exposure and DNA damage (Bonilla et al, 2016, Long et al, 2023). Unlike older populations, where incidence is typically higher in men, women aged 20–44 years had higher ASIRs of melanoma and BCC, consistent with a 2005 *JAMA* report (Christenson et al, 2005, Wang et al, 2025). Despite a higher incidence in women, mortality was greater in men, a pattern also confirmed in CDC WONDER data. Men are often diagnosed at a more advanced stage with greater tumor thickness, linked to lower health-seeking behavior (Joosse et al, 2011, Olsen et al, 2025). Women may possess a prognostic advantage from stronger immune responses and a favorable hormonal profile (Klein and Flanagan, 2016, Schwartz et al, 2019). These clinical and biological factors, combined with behavioral differences such as greater occupational sun exposure and lower sunscreen use among men, likely converge to explain their higher mortality (Paul et al, 2024).

Significant geographic and socioeconomic disparities also persist. High-SDI countries such as the United States, Australia, and New Zealand bear the highest burden but have also achieved significant reductions in melanoma mortality owing to robust healthcare systems (Arnold et al, 2022). However, our analysis of United States data reveals a critical urban–rural gap, with a slower, more volatile decline in mortality and DALYs in nonmetropolitan areas, suggesting challenges in primary care access and referral pathways. For regions with rapidly rising incidence, such as East Asia, strengthening primary prevention is crucial.

Decomposition analysis identified population growth as the main driver of the rising burden of skin cancer in younger adults. Complementing Nordpred’s conservative trend dampening with Bayesian age-period-cohort’s use of temporal borrowing to capture recent surges (particularly in NMSC), we bracket plausible future trajectories. Projections to 2040 suggest continued global increases in NMSC, contrasted with declining melanoma incidence. Although the overall burden in young adults is lower than in older populations, the rising absolute numbers, particularly the rapid increases in some regions, represent a critical and overlooked public health challenge. Compared with intervention in older patients, early intervention in younger adults yields superior survival outcomes and reduces future disease burden in later life (Wang et al, 2025). In advanced-stage disease, younger adults may paradoxically have poorer outcomes than older patients (Wojcik et al, 2023). Incorporating younger adults into global skin cancer control strategies, with dedicated emphasis on early screening and risk identification, is essential to curb future burden and reduce health inequalities.

The global burden of skin cancer in young adults is rising, characterized by a decrease in melanoma but an increase in NMSCs. This trend establishes a clear mandate to target this historically overlooked population. Considering their unique clinical paradox—superior survival with early detection, yet poorer outcomes in advanced stages—prioritizing enhanced early screening and risk-based management is a critical, high-yield strategy to curb the future population-wide burden of skin cancer.

## Materials and Methods

### Data sources

“Younger adults” (aged 20–44 years) were defined per the relevant adolescent and young adult oncology literature (Aldhaleei et al, 2024, Fidler et al, 2017, Weir et al, 2008). The GBD 2021 study is a comparative epidemiological framework designed to generate internally consistent estimates of fatal and nonfatal health loss across time, age, sex, and geography. It provides estimates for 371 diseases and injuries in 204 countries and territories and 811 subnational locations from 1990 to 2021. Rather than a single cohort with a fixed sample size, GBD synthesizes multiple population-level data sources, including vital registration systems, censuses, household surveys, cancer registries, verbal autopsy data, and administrative records, using standardized modeling approaches (Brauer et al, 2024, Naghavi et al, 2024). For this study, we extracted estimates for the five 5-year age groups spanning 20–44 years (20–24, 25–29, 30–34, 35–39, and 40–44 years) and summarized incidence, prevalence, deaths, DALYs, and corresponding ASRs for melanoma, BCC, and SCC, with 95% uncertainty intervals. In GBD, both melanoma and NMSC groupings include International Classification of Diseases, Tenth Revision codes for in situ and uncertain-behavior lesions, which cannot be separated in the public data. All GBD ASRs reported in this study were obtained from GBD 2021 and were calculated using the GBD standard population structure.

United States mortality trends (1999–2023) were assessed using CDC WONDER. Analyses of younger adults were restricted to the 2 standard 10-year age groups (25–34 and 35–44 years), which together constitute the 25–44-year-old population. For the overall 25–44-year analyses, age-adjusted mortality rates were obtained directly from CDC WONDER; these rates are standardized by the National Center for Health Statistics to the projected year 2000 United States standard population using the direct method. For age-stratified analyses (25–34 and 35–44 years), CDC WONDER does not calculate age-adjusted rates when data are stratified by age; therefore, these strata are reported as age-specific crude mortality rates. Underlying-cause deaths were defined by International Classification of Diseases, Tenth Revision codes C43 (malignant melanoma of skin), C44 (other malignant neoplasms of skin), and C84 (peripheral and cutaneous T-cell lymphomas). In situ neoplasms are rarely recorded as the underlying cause of death, and cell counts <10 are suppressed; accordingly, mortality data largely reflect invasive disease.

This study was a population-level observational analysis of secondary data derived from GBD 2021 and CDC WONDER. Accordingly, we reported the study in accordance with the Strengthening the Reporting of Observational Studies in Epidemiology statement, as applicable (von Elm et al, 2007). Forecasting analyses were separately described in the Statistical Analysis section. Because the analysis relied exclusively on publicly available, deidentified, aggregate data and did not involve direct contact with human participants or access to identifiable private information, institutional review board approval and informed consent were not required.

### Modeling of trends, disparities, and future burden

Temporal trends were assessed using joinpoint regression to identify inflection points and calculate the annual percent change for each segment (Clegg et al, 2009, Kim et al, 2000). The average annual percent change was calculated as a summary measure for the overall period. Frontier analysis (data envelopment analysis) was used to benchmark the potential for reducing the skin cancer burden, and decomposition analysis was applied to quantify the relative contributions of demographic and epidemiological factors (Barber et al, 2017, Xie et al, 2018). Global health inequalities were measured using the slope index of inequality for absolute disparities and the concentration index for relative disparities. Future incidence rates through 2040 were projected using 2 complementary models: a Bayesian age-period-cohort model and the Nordpred model (Arnold et al, 2020).

### Statistical analysis

ASRs per 100,000 persons were used to compare the skin cancer burden across regions and over time. The SDI is a composite measure of sociodemographic development, derived from the geometric mean of 0-to-1 rescaled values of lag-distributed income per capita, mean educational attainment among individuals aged ≥15 years, and the total fertility rate under age 25 years. Temporal trends in ASRs were quantified using the EAPC, derived from a linear regression of the natural logarithm of ASR on calendar year. Trends were classified as increasing if both the EAPC estimate and its 95% CI were positive, decreasing if both were negative, and stable otherwise. For CDC WONDER analyses, joinpoint models were applied to age-adjusted mortality rates for the overall 25–44-year population and subgroups defined by sex, region, race, and urbanization and to crude age-specific mortality rates for the 25–34-year and 35–44-year strata. Analyses were conducted using R (version 4.4.0), Python (version 3.9), and Joinpoint (version 5.4.0). A 2-sided *P* < .05 was considered statistically significant.

## Ethics Statement

This study was based exclusively on publicly available, deidentified, aggregate secondary data from Global Burden of Disease 2021 and Centers for Disease Control and Prevention Wide-ranging Online Data for Epidemiologic Research. According to institutional policy at Leiden University Medical Center, this type of study does not require institutional review board approval or informed consent.

## Data Availability Statement

The data supporting this study are publicly available from the Global Burden of Disease Results Tool (https://vizhub.healthdata.org/gbd-results/) and Centers for Disease Control and Prevention Wide-ranging Online Data for Epidemiologic Research mortality databases, including the Underlying Cause of Death database (https://wonder.cdc.gov/). The corresponding author, AEG, can be contacted at a.e.l.ghalbzouri@lumc.nl for assistance with data access and analytic details upon reasonable request.

## ORCIDs

Zhiqiang Ma: http://orcid.org/0009-0007-0890-4222

Pingyu An: http://orcid.org/0009-0005-2564-6750

Shidi Wu: http://orcid.org/0000-0001-8192-8123

Remco van Doorn: http://orcid.org/0000-0002-7019-6673

Maarten Vermeer: http://orcid.org/0000-0002-5872-4613

Abdoelwaheb El Ghalbzouri: http://orcid.org/0000-0001-8351-7715

## Conflict of Interest

The authors state no conflict of interest.

## References

[bib1] Aggarwal P., Knabel P., Fleischer A.B. (2021). United States burden of melanoma and non-melanoma skin cancer from 1990 to 2019. J Am Acad Dermatol.

[bib2] Aldhaleei W.A., Wallace M.B., Bhagavathula A.S. (2024). Trends and age-period-cohort effect on the incidence of early-onset colorectal cancer (20-44 years) from 1990 to 2021 in the United States. Cancers (Basel).

[bib46] Armstrong B.K., Cust A.E. (2017). Sun exposure and skin cancer, and the puzzle of cutaneous melanoma: A perspective on Fears et al. Mathematical models of age and ultraviolet effects on the incidence of skin cancer among whites in the United States, American Journal of Epidemiology 1977; 105: 420-427. Cancer Epidemiol.

[bib4] Arnold M., Park J.Y., Camargo M.C., Lunet N., Forman D., Soerjomataram I. (2020). Is gastric cancer becoming a rare disease? A global assessment of predicted incidence trends to 2035. Gut.

[bib5] Arnold M., Singh D., Laversanne M., Vignat J., Vaccarella S., Meheus F. (2022). Global burden of cutaneous melanoma in 2020 and projections to 2040. JAMA Dermatol.

[bib6] Barber R.M., Fullman N., Sorensen R.J.D., Bollyky T., McKee M., Nolte E. (2017). Healthcare Access and Quality Index based on mortality from causes amenable to personal health care in 195 countries and territories, 1990–2015: a novel analysis from the Global Burden of Disease Study 2015. Lancet.

[bib7] Bonilla X., Parmentier L., King B., Bezrukov F., Kaya G., Zoete V. (2016). Genomic analysis identifies new drivers and progression pathways in skin basal cell carcinoma. Nat Genet.

[bib8] Brauer M., Roth G.A., Aravkin A.Y., Zheng P., Abate K.H., Abate Y.H. (2024). Global burden and strength of evidence for 88 risk factors in 204 countries and 811 subnational locations, 1990–2021: a systematic analysis for the Global Burden of Disease Study 2021. Lancet.

[bib9] Carlino M.S., Larkin J., Long G.V. (2021). Immune checkpoint inhibitors in melanoma. Lancet.

[bib10] Christenson L.J., Borrowman T.A., Vachon C.M., Tollefson M.M., Otley C.C., Weaver A.L. (2005). Incidence of basal cell and squamous cell carcinomas in a population younger than 40 years. Jama.

[bib11] Clegg L.X., Hankey B.F., Tiwari R., Feuer E.J., Edwards B.K. (2009). Estimating average annual per cent change in trend analysis. Stat Med.

[bib12] Close A.G., Dreyzin A., Miller K.D., Seynnaeve B.K.N., Rapkin L.B. (2019). Adolescent and young adult oncology-past, present, and future. CA Cancer J Clin.

[bib13] Cullen J.K., Simmons J.L., Parsons P.G., Boyle G.M. (2020). Topical treatments for skin cancer. Adv Drug Deliv Rev.

[bib14] Curti B.D., Faries M.B. (2021). Recent advances in the treatment of melanoma. N Engl J Med.

[bib15] Del Mar C. (1995). Slip, slop, slap and wrap. Should we do more to prevent skin cancer?. Med J Aust.

[bib16] Dummer R., Vermeer M.H., Scarisbrick J.J., Kim Y.H., Stonesifer C., Tensen C.P. (2021). Cutaneous T cell lymphoma. Nat Rev Dis Primers.

[bib17] El Ghissassi F., Baan R., Straif K., Grosse Y., Secretan B., Bouvard V. (2009). A review of human carcinogens--part D: radiation. Lancet Oncol.

[bib18] Fidler M.M., Gupta S., Soerjomataram I., Ferlay J., Steliarova-Foucher E., Bray F. (2017). Cancer incidence and mortality among young adults aged 20-39 years worldwide in 2012: a population-based study. Lancet Oncol.

[bib19] Fitzmaurice C., Abate D., Abbasi N., Abbastabar H., Abd-Allah F., Abdel-Rahman O. (2019). Global, regional, and national cancer incidence, mortality, years of life lost, years lived with disability, and disability-adjusted life-years for 29 cancer groups, 1990 to 2017: a systematic analysis for the global burden of disease study. JAMA Oncol.

[bib20] Garbe C., Forsea A.M., Amaral T., Arenberger P., Autier P., Berwick M. (2024). Skin cancers are the most frequent cancers in fair-skinned populations, but we can prevent them. Eur J Cancer.

[bib21] Joosse A., de Vries E., Eckel R., Nijsten T., Eggermont A.M., Hölzel D. (2011). Gender differences in melanoma survival: female patients have a decreased risk of metastasis. J Invest Dermatol.

[bib22] Kim H.J., Fay M.P., Feuer E.J., Midthune D.N. (2000). Permutation tests for joinpoint regression with applications to cancer rates. Stat Med.

[bib23] Klein S.L., Flanagan K.L. (2016). Sex differences in immune responses. Nat Rev Immunol.

[bib24] Leeneman B., Schreuder K., Uyl-de Groot C.A., van Akkooi A.C.J., Haanen J., Wakkee M. (2021). Stage-specific trends in incidence and survival of cutaneous melanoma in the Netherlands (2003-2018): a nationwide population-based study. Eur J Cancer.

[bib25] Long G.V., Swetter S.M., Menzies A.M., Gershenwald J.E., Scolyer R.A. (2023). Cutaneous melanoma. Lancet.

[bib26] Miller K.D., Fidler-Benaoudia M., Keegan T.H., Hipp H.S., Jemal A., Siegel R.L. (2020). Cancer statistics for adolescents and young adults, 2020. CA Cancer J Clin.

[bib27] Naghavi M., Ong K.L., Aali A., Ababneh H.S., Abate Y.H., Abbafati C. (2024). Global burden of 288 causes of death and life expectancy decomposition in 204 countries and territories and 811 subnational locations, 1990–2021: a systematic analysis for the Global Burden of Disease Study 2021. The Lancet.

[bib28] Olsen C.M., Pandeya N., Miranda-Filho A., Rosenberg P.S., Whiteman D.C. (2025). Does sex matter? Temporal analyses of melanoma trends among men and women suggest etiologic heterogeneity. J Invest Dermatol.

[bib29] Paul S., Chen Y., Mohaghegh M. (2024). Analysis of prevalence, socioeconomic and disease trends of non-melanoma skin cancer in New Zealand from 2008 to 2022. J Epidemiol Glob Health.

[bib30] Reiter O., Mimouni I., Gdalevich M., Marghoob A.A., Levi A., Hodak E. (2019). The diagnostic accuracy of dermoscopy for basal cell carcinoma: a systematic review and meta-analysis. J Am Acad Dermatol.

[bib31] Schadendorf D., van Akkooi A.C.J., Berking C., Griewank K.G., Gutzmer R., Hauschild A. (2018). Melanoma. Lancet.

[bib32] Schmidt C.W. (2012). UV radiation and skin cancer: the science behind age restrictions for tanning beds. Environ Health Perspect.

[bib33] Schwartz M.R., Luo L., Berwick M. (2019). Sex differences in melanoma. Curr Epidemiol Rep.

[bib34] von Elm E., Altman D.G., Egger M., Pocock S.J., Gøtzsche P.C., Vandenbroucke J.P. (2007). The strengthening the reporting of observational studies in epidemiology (STROBE) statement: guidelines for reporting observational studies. Ann Intern Med.

[bib35] Wang R., Chen Y., Shao X., Chen T., Zhong J., Ou Y. (2025). Burden of skin cancer in older adults from 1990 to 2021 and modelled projection to 2050. JAMA Dermatol.

[bib36] Waseh S., Lee J.B. (2023). Advances in melanoma: epidemiology, diagnosis, and prognosis. Front Med (Lausanne).

[bib37] Watts C.G., Drummond M., Goumas C., Schmid H., Armstrong B.K., Aitken J.F. (2018). Sunscreen use and melanoma risk among young Australian adults. JAMA Dermatol.

[bib38] Weir H.K., Jim M.A., Marrett L.D., Fairley T. (2008). Cancer in American Indian and Alaska Native young adults (ages 20-44 years): US, 1999-2004. Cancer.

[bib39] Whiteman D.C., Neale R.E., Baade P., Olsen C.M., Pandeya N. (2024). Changes in the incidence of melanoma in Australia, 2006-2021, by age group and ancestry: a modelling study. Med J Aust.

[bib40] Williams M.S., Buhalog B., Blumenthal L., Stratman E.J. (2018). Tanning salon compliance rates in states with legislation to protect youth access to UV tanning. JAMA Dermatol.

[bib41] Wojcik K.Y., Hawkins M., Anderson-Mellies A., Hall E., Wysong A., Milam J. (2023). Melanoma survival by age group: population-based disparities for adolescent and young adult patients by stage, tumor thickness, and insurance type. J Am Acad Dermatol.

[bib42] Wysong A. (2023). Squamous-cell carcinoma of the skin. N Engl J Med.

[bib43] Xie Y., Bowe B., Mokdad A.H., Xian H., Yan Y., Li T. (2018). Analysis of the Global Burden of Disease study highlights the global, regional, and national trends of chronic kidney disease epidemiology from 1990 to 2016. Kidney Int.

[bib44] Yélamos O., Braun R.P., Liopyris K., Wolner Z.J., Kerl K., Gerami P. (2019). Usefulness of dermoscopy to improve the clinical and histopathologic diagnosis of skin cancers. J Am Acad Dermatol.

[bib45] Zhou L., Zhong Y., Han L., Xie Y., Wan M. (2025). Global, regional, and national trends in the burden of melanoma and non-melanoma skin cancer: insights from the global burden of disease study 1990-2021. Sci Rep.

